# Comparison of library preparation methods reveals their impact on interpretation of metatranscriptomic data

**DOI:** 10.1186/1471-2164-15-912

**Published:** 2014-10-20

**Authors:** Adriana Alberti, Caroline Belser, Stéfan Engelen, Laurie Bertrand, Céline Orvain, Laura Brinas, Corinne Cruaud, Laurène Giraut, Corinne Da Silva, Cyril Firmo, Jean-Marc Aury, Patrick Wincker

**Affiliations:** CEA-Institut de Génomique, Genoscope, Centre National de Séquençage, 2 rue Gaston Crémieux, CP5706 F-91057, Evry Cedex, France; Université d’Evry, UMR 8030, CP5706 Evry, France; Centre National de la Recherche Scientifique (CNRS), UMR 8030, CP5706 Evry, France

**Keywords:** Metatranscriptomics, cDNA synthesis method, Gene expression

## Abstract

**Background:**

Metatranscriptomics is rapidly expanding our knowledge of gene expression patterns and pathway dynamics in natural microbial communities. However, to cope with the challenges of environmental sampling, various rRNA removal and cDNA synthesis methods have been applied in published microbial metatranscriptomic studies, making comparisons arduous. Whereas efficiency and biases introduced by rRNA removal methods have been relatively well explored, the impact of cDNA synthesis and library preparation on transcript abundance remains poorly characterized. The evaluation of potential biases introduced at this step is challenging for metatranscriptomic samples, where data analyses are complex, for example because of the lack of reference genomes.

**Results:**

Herein, we tested four cDNA synthesis and Illumina library preparation protocols on a simplified mixture of total RNA extracted from four bacterial species. In parallel, RNA from each microbe was tested individually. cDNA synthesis was performed on rRNA depleted samples using the TruSeq Stranded Total RNA Library Preparation, the SMARTer Stranded RNA-Seq, or the Ovation RNA-Seq V2 System. A fourth experiment was made directly from total RNA using the Encore Complete Prokaryotic RNA-Seq. The obtained sequencing data were analyzed for: library complexity and reproducibility; rRNA removal efficiency and bias; the number of genes detected; coverage uniformity; and the impact of protocols on expression biases. Significant variations, especially in organism representation and gene expression patterns, were observed among the four methods. TruSeq generally performed best, but is limited by its requirement of hundreds of nanograms of total RNA. The SMARTer method appears the best solution for smaller amounts of input RNA. For very low amounts of RNA, the Ovation System provides the only option; however, the observed biases emphasized its limitations for quantitative analyses.

**Conclusions:**

cDNA and library preparation methods may affect the outcome and interpretation of metatranscriptomic data. The most appropriate method should be chosen based on the available quantity of input RNA and the quantitative or non-quantitative objectives of the study. When low amounts of RNA are available, as in most metatranscriptomic studies, the SMARTer method seems to be the best compromise to obtain reliable results. This study emphasized the difficulty in comparing metatranscriptomic studies performed using different methods.

**Electronic supplementary material:**

The online version of this article (doi:10.1186/1471-2164-15-912) contains supplementary material, which is available to authorized users.

## Background

Metatranscriptomic approaches based on next generation sequencing have emerged as a powerful tool to provide insight into microbial activity in complex environmental communities, such as marine [1–7], soil [8–10] or human internal organ ecosystems [11]. However, these studies have used many different methods for cDNA synthesis, making comparison of the results difficult and highlighting the challenges of analyzing RNA samples from environmental communities. Indeed, due to sampling constraints, the quality and quantity of RNA available for library preparation may be very heterogeneous and limited [12, 13]. For these reasons, the most common strategies used for cDNA synthesis rely mainly on polyadenylation of prokaryotic mRNA, followed by a linear or exponential amplification of cDNA [1–7, 11]. Usually, an rRNA removal treatment precedes the cDNA synthesis, to reduce the predominant rRNA fraction (which can be more than 95%) [14]. Different methods for ribosomal RNA removal have been reported [15–17] and compared in GC-rich transcriptomes [18], and in synthetic [19] and natural microbial communities [20, 21], giving an extensive overview on their efficiency and biases. By contrast, even if most studies validate the transcript abundance inferred by sequencing data using quantitative real-time reverse transcription PCR (qRT-PCR) to check for artefacts in cDNA amplification [1–4], the bias introduced at this step remains poorly explored.

Recently, several new cDNA preparation methods especially conceived for RNA-Seq have been released and, for some of them, the impact on data analysis and interpretation in eukaryotic transcriptomes has been reported [22–24]. Our work aimed to expand upon these studies by focusing on microbial metatranscriptomics. We tested three methods that should, in principle, permit the experiment to be initiated from small inputs, equal to or lower than 100 ng total RNA, a realistic quantity available from environmental sampling. We prepared libraries with SMARTer Stranded RNA-Seq Kit from Clontech (SMART), the Ovation RNA-Seq System V2 (OV) and the Encore Complete Prokaryotic RNA-Seq System (ENC, recently renamed Ovation Complete Prokaryotic RNA-Seq) from NuGEN and compared them with the TruSeq Stranded Total RNA Library Preparation from Illumina (TS), one of the most widely applied methods for RNA-Seq studies (for a methods overview, see Table 1). Whereas TS and SMART cDNA protocols are based on synthesis by random priming after RNA fragmentation, the ENC synthesis is carried out using selective primers with decreased affinity for rRNA sequences, with no need for prior ribosomal depletion. Finally, the OV system allows cDNA synthesis by oligo(dT) and random priming. Even if this protocol is not especially developed for prokaryotic RNA-Seq, it proved to be very efficient to detect viral RNA sequences in clinical samples containing ultra-low amounts (some femtograms) of viral RNA genomes [25]. Therefore, we chose to test it because of its robustness in library yield when very low amounts of RNA are available. This is possible because of a linear amplification step that allows the production of micrograms of double-stranded cDNA from a few nanograms of RNA.

**Table 1:** **Summary of the principal characteristics of the RNA-Seq library preparation kits evaluated in this study**

	TruSeq stranded	Encore complete	Ovation RNA-Seq V2	SMARTer stranded
**RNA input (minimal requirement according to the manufacturer)**	100 ng depleted RNA	100 ng total RNA	0,5 ng depleted RNA	1 ng depleted RNA
**Minimum quality**	FFPE	RIN >7	FFPE	FFPE
**rRNA depletion required**	Yes	No	Yes	Yes
**cDNA synthesis**	Random primers	Selective priming	Random and oligo(dT) primers	Random primers
**Fragmentation method**	RNA by divalent cations + heat	cDNA by Covaris shearing	cDNA by Covaris shearing	RNA by heat
**Strand selection**	Yes	Yes	No	Yes
**Library preparation method and reagents**	Included	Included	Not included	Included
**Multiplex capacity**	96-plex	16-plex	according to the library preparation method chosen	12-plex
**Experiment duration**	6 hours	7 hours	4.5 hours for cDNA synthesis	4.5 hours
			+ time for library preparation	

FFPE: Formalin-fixed, paraffin-embedded tissue.

RIN: RNA integrity number.

In this study, we tested the four RNA-Seq methods on four well-known microbial species, individually and pooled together, and assessed their performances. We evaluated rRNA depletion efficiency and bias; library complexity; alignment efficiency; gene detection sensitivity and abundance; evenness of transcript coverage; and technical reproducibility. Our results revealed the strengths and weaknesses of each method, and provided guidelines for choosing the method that is best adapted for metatranscriptomic studies.

## Results

### Experimental design

To overcome the extreme complexity of environmental samples, we set up a simplified benchmark comprising total RNA from four microbial species: two Gram negative organisms, *Escherichia coli* MG1655 and *Acinetobacter baylyi* ADP1; and two Gram positive organisms, *Lactococcus lactis* MG1363 and *Bacillus subtilis* 168. The four species, for which reference genomes are available, have different GC contents, heterogeneous genome sizes and gene contents (Additional file 1: Table S1). They were cultivated in different conditions reflecting their different gene expression dynamics.

We prepared libraries using total RNA (control libraries) and depleted RNA individually from the four microorganisms and from a pool of four RNAs (hereafter named MIX) in duplicate, starting from the same individual RNAs and MIX sample. Using Illumina paired-end sequencing, we generated, on average, 20.5 M reads and 0.8 M reads for the control and depleted RNA libraries, respectively (Table 2). Reads were cleaned as described in the Methods and subsequent analyses were performed on the remaining data (cleaned reads). Here we discuss the data analyses of the *L. lactis* and MIX datasets. The results obtained for the other bacterial species are mostly presented in the Additional files.

**Table 2:** **Sequences and mapping statistics**

	Raw reads (millions)		% rRNA^a^		Cleaned reads^b^(millions)		% mapped reads^c^		% duplication rates^d^	
Library name	replicate	replicate	replicate	replicate	replicate	replicate	replicate	replicate	replicate	replicate
	1	2	1	2	1	2	1	2	1	2
**TS_L.L**	0.724	0.747	0.14	0.23	0.720	0.743	77.2	72.3	2.19	5.84
**ENC_L.L**	0.764	0.739	36.6	37.96	0.481	0.454	67.1	51.2	5.52	13
**OV_L.L**	0.809	0.792	0.2	0.39	0.802	0.784	65.3	61	2.3	2.45
**SMART_L.L**	0.904	0.697	2.9	2.88	0.837	0.650	62.9	63.7	8.76	7.62
**TS_L.L control**	22.161	19.845	95.6	95.4	0.960	0.885	71.2	71.4	8.47	6.92
**OV_L.L control**	21.989	17.513	71	78.,5	6.302	3.676	73	74.4	1.01	0.57
**SMART_L.L control**	24.806	18.798	95	95.1	1.146	0.831	51.5	51.9	54	61.6
**TS_MIX**	0.754	0.769	0.21	2.27	0.748	0.747	73.1	72.1	1.23	1.59
**ENC_MIX**	0.749	0.732	37	36.61	0.470	0.461	66.5	66	7.4	5.58
**OV_MIX**	0.864	1.177	0.9	0.6	0.849	1.161	55.1	54.7	0.93	0.85
**SMART_MIX**	0.824	0.726	0.77	0.96	0.809	0.702	65.7	66.9	6.5	7.03
**TS_MIX control**	20.257	30.693	93	94.1	1.393	1.775	70.4	71.1	3.24	3.,28
**OV_MIX control**	19.510	17.984	84	82	2.983	3.215	75.2	70.4	1.78	0.39
**SMART_MIX control**	14.699	18.603	93.5	93.5	0.915	1.160	63.9	61.9	23	39

_L.L: library prepared from *L. lactis* depleted RNA.

_L.L control: library prepared from *L. lactis* total RNA.

_MIX: library prepared from the MIX depleted RNA.

_MIX control: library prepared from the MIX total RNA.

^a^proportion of rRNA reads detected in the raw reads.

^b^number of sequences remaining after the data quality control pipeline treatment applied on raw reads.

^c^proportion of cleaned reads uniquely mapped on CDS sequences.

^d^duplication rate estimated on 100 000 cleaned reads.

### rRNA removal efficiency

rRNA depletion using the Ribo-Zero kit was efficient: the proportion of rRNA reads was less than 1% for the TS and OV libraries, and less than 3% for the SMART library (Table 2). By contrast, this proportion increased to 38% in the ENC libraries, suggesting that the selective priming in the ENC method is not efficient. This led to a decreased number of useful reads, which could negatively affect metatranscriptomic studies where deep sequencing is needed to obtain sufficient coverage.

### Library complexity

We assessed the complexity of each library by calculating the number of duplicated paired reads. We preferred to evaluate the duplication on paired reads rather than on single reads, as they take into account the PCR duplicates. The results (Table 2) showed satisfactory values for all experiments, except for the SMART control libraries. The very high duplication rate in these libraries could reflect an insufficient amount of input RNA (1 ng), which resulted in a low final library yield. Consequently, the number of useful reads in these libraries was lower. The duplication rates in the OV and ENC libraries were not comparable to the others, because in these preparations, cDNAs are randomly sheared after their synthesis. Indeed, this fragmentation step introduces additional diversity into the starting position of the sequence.

### Transcript coverage and orientation

Reads from the *L. lactis* libraries were aligned against the *L. lactis* CDS sequences to estimate the specificity of each method. TS produced the best mapping percentages to the CDS sequences (72 and 77%), followed by OV and SMART (61 to 65%) (Table 2). Mapping rates were variable for the ENC samples (51 and 67%), indicating that this protocol was less reproducible.

Based on *L. lactis* gene prediction, we assessed the orientation of the reads in SMART, ENC and TS libraries, which were expected to preserve the coding strand information. Around 99% of SMART and ENC reads mapped to the sense strand, whereas around 99% of TS reads mapped to the antisense strand, as expected according to the manufacturer’s specifications. These strand-specific methods are advantageous for *de novo* transcriptome assembly, gene annotation and detection of potential antisense transcription.

The ENC protocol had the highest rate of intergenic covered bases (21%) and OV had the lowest (13%) (Additional file 1: Table S2). As a rigorous DNAse treatment was applied, we could infer that the presence of reads mapping to intergenic regions reflected the capture of RNAs at different steps of their maturation or synthesis. Indeed, the same RNA extractions were used for each experiment, suggesting that these differences in the percentages of intergenic regions could be attributed to the different RNA reverse transcription protocols.

**Figure 1:**
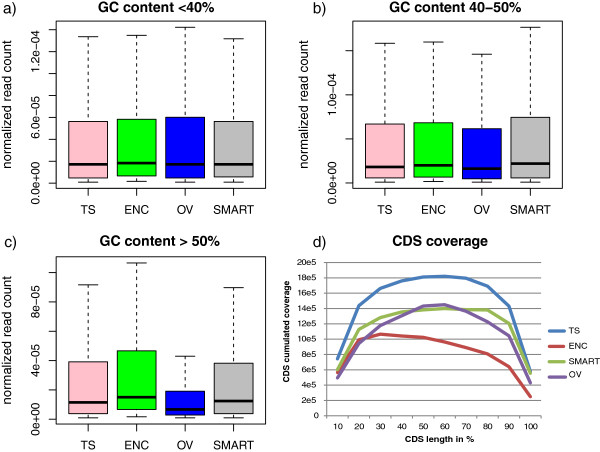
**CDS coverage and GC content. (a)**, **(b)**, **(c)**: box plots distribution of the CDS read counts normalized by the total read count for three categories of GC content (<40%, 40-50% and >50% GC) and for each MIX library. **(d)**: distribution of the cumulated coverage along the length of the annotated CDS for MIX libraries (in 5′- > 3′ orientation).

We evaluated the evenness of CDS coverage for each method, based on the *L. lactis* CDS coverage extracted from the MIX experiments. TS, OV and SMART covered the CDS globally over their entire lengths, with a drop at the 5′ and 3′ extremities. ENC had a higher coverage rate at the 5′ extremities. Plots of CDS-accumulated coverage versus their GC content showed that high GC content CDS were detected less frequently in OV libraries, whereas the other methods presented the same global profile for all the CDS, irrespective of GC content (Figure 1).

### Metatranscriptome bias evaluation

The MIX samples were especially useful for assessing if the cDNA and library preparation methods had introduced bias into the pattern of gene expression from each bacterial species in a multispecies context. After alignment of MIX library reads against CDSs, we observed that the distribution of reads attributed to each organism was similar for the TS, SMART and ENC samples (Additional file 1: Table S3). However, the distribution of the OV reads was quite different: in particular, 38% of the reads were attributed to *L. lactis*, compared with 20–25% for the other methods. Again, the OV method seemed to introduce a bias in relation to the GC content of the genomes (Additional file 1: Table S1).

We then normalized the RNA-Seq fragment counts by calculating the fragments per kilobase of CDS per million fragments mapped (FPKM) and performed a linear correlation between the *L. lactis* and MIX samples. *L. lactis* mRNA abundances correlated well in the MIX and the single species samples for all the methods, except for SMART (Table 3 and Additional file 2: Figure S1). For this method, however, almost all the CDSs in the *L. lactis* sample were present in the MIX. Indeed, we observed that absent genes in the MIX had the lowest FPKM values in the *L. lactis* sample. The *L. lactis* RNA quantity used in the MIX sample preparations was four-fold less than that in the *L. lactis* libraries. We inferred that this led to a loss of information, essentially for the lowest-expressed genes.

**Table 3:** **Pearson correlation coefficients**

		***L. lactis***vs MIX^a^	Replicates^b^	Other methods vs TS^c^	Depleted RNA vs total RNA^d^
***L. lactis***	TS	0.992	0.886		0.913
	ENC	0.985	0.736	0.570	
	OV	0.974	0.995	0.696	0.971
	SMART	0.753	0.995	0.805	0.9
**MIX**	TS		0.958		0.884
	ENC		0.980	0.498	
	OV		0.998	0.589	0.911
	SMART		0.989	0.772	0.924

Pearson correlation coefficients between:

^a^the *L. lactis* library and the MIX library for each method.

^b^the two replicates for each experiment.

^c^ENC, OV or SMART *L. lactis* libraries and TS *L. lactis* libraries.

^d^the depleted RNA library and the total RNA library for each method.

### Experimental reproducibility

To test the robustness of each method, the Pearson correlation coefficients between the two replicates of the *L. lactis* samples were calculated (Table 3). The OV and SMART methods performed best (Pearson correlation coefficient r =0.995), whereas the ENC method was the least reproducible (r =0.736) (Table 3 and Additional file 2: Figure S2). The variability in the CDS detection between two replicates was around 8%, except for ENC (11%). For the MIX samples, replicates for each of the four methods all correlated well (Additional file 2: Figure S3).

### Gene expression level

We compared the gene expression patterns to examine how they were affected by the different cDNA synthesis methods. We considered the TS experiments as a reference and calculated the Pearson correlation and the percentage of differentially expressed genes (DEGs) for each method in the *L. lactis* samples (Tables 3 and 4, Additional file 2: Figures S4 and S5). This choice was based on the fact that TruSeq experiments are not made with limited amounts of RNA and should better reflect the complexity of the transcriptome. Indeed, in this study, experiments performed with TS produced the best results in terms of library complexity, gene detection and coverage, and reproducibility.

**Table 4:** **Proportions of genes detected as differentially expressed**

	***L. lactis***vs***L. lactis***TS^a^	MIX vs TS_MIX^b^	***L. lactis***depleted RNA vs ***L. lactis***total RNA^c^	MIX depleted RNA vs MIX total RNA^d^
TS			6.2	4.7
ENC	30.8	32.6		
OV	27.6	35.4	8	14.5
SMART	2.2	9.2	21.3	1.1

Proportions of genes detected as differentially expressed between:

^a^ENC, OV or SMART *L. lactis* library and TS *L. lactis* library.

^b^ENC, OV or SMART MIX library and TS MIX library.

^c^TS,OV or SMART *L. lactis* depleted RNA library and TS,OV or SMART *L. lactis* total RNA library.

^d^TS,OV or SMART MIX depleted RNA library and TS,OV or SMART MIX total RNA library.

**Figure 2:**
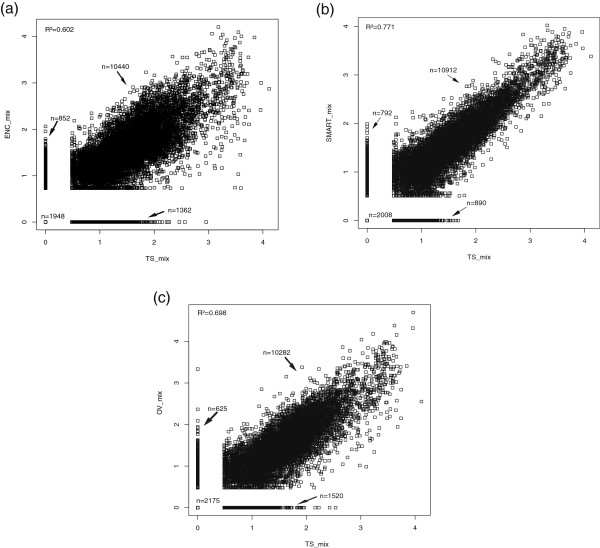
**Gene expression profile of TS versus OV, SMART and ENC methods in MIX samples.** Gene expression profile from TS_MIX versus **(a)** ENC_MIX **(b)** SMART_MIX **(c)** OV_MIX samples. This figure shows the log scatter plots and the coefficients of determination (R^2^) obtained by comparing FPKM values for 14602 annotated CDS in the mix of bacteria.

Surprisingly, broad differences in gene expression patterns were observed. ENC was the least correlated method (r =0.57), with 31% of *L. lactis* genes detected as differentially expressed (p < 0.01). The OV experiments performed slightly better (r =0.696) with 28% of DEGs. SMART was the best correlated method (r =0.805) and the number of genes detected as differentially expressed in comparison to the TS experiment was not significant. The same comparison made within the MIX samples showed similar results. The SMART MIX sample was the best correlated, even if the percentage of DEGs was slightly higher (9.2%) than in the *L. lactis* libraries (Table 4, Figure 2 and Additional file 2: Figure S6).

**Figure 3:**
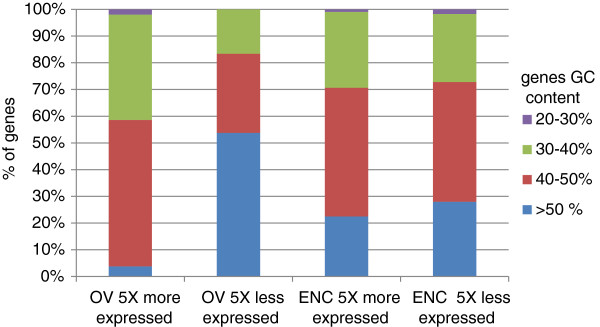
**Evaluation of the GC bias in the differential expression analysis.** Genes detected as the most differentially expressed were extracted (5 fold more or less differentially expressed) for ENC and OV MIX libraries. The proportions of CDS for different GC content intervals were plotted to evaluate the bias introduced by the cDNA synthesis method.

We showed previously that the GC content could influence the results. Therefore, we verified if highly differentially expressed genes were correlated with their GC content. This analysis was limited to ENC and OV MIX libraries as the number of genes to be considered in the SMART libraries was not statistically significant. The results showed that the ENC profiles of overexpressed and underexpressed genes were similar (Figure 3). No correlation was evident between the GC content of the genes and their differential expression. In contrast, differences in OV gene expression patterns were clearly correlated with the GC content. Specifically, expression of low GC content genes was overestimated and GC-rich genes were penalized, confirming our previous observations that the OV method introduces a bias in favor of low GC genes.

To validate the results of the sequencing experiments, we performed qRT-PCR analysis on certain selected *L. lactis* genes and compared qRT-PCR quantifications with the read counts of the same genes in the sequenced transcriptomes. A strong positive correlation was observed for the TS and SMART experiments (r =0.948 and 0.965, respectively). ENC showed a slightly lower correlation (r =0.899), whereas gene expression levels in OV were significantly different (r =0.603) (Additional file 2: Figure S7). This experiment confirms that, among the “low-input” methods, SMART most reliably represents the mRNA abundance.

### Depletion effect

Ribo-Zero treatment protocol was adapted for SMART and OV experiments to fit with total RNA inputs of 50 ng. Hence, we investigated if this technical modification had an impact on the mRNA expression profiles. Linear correlation of gene expression patterns between libraries prepared from total RNA (control libraries) or depleted ones showed similar percentages of mapped reads (Table 2), as well as a strong correlation (Table 3) for TS (r =0.913) and OV (r =0.97). No bias in the expression profiles was observed (Table 4 and Additional file 2: Figures S8 and S9).

Curiously, we noticed that in the OV control libraries, the percentage of rRNA reads was significantly lower than expected (less than 78.5% and 84% in the *L. lactis* and MIX libraries, respectively). This observation suggested that the OV RNA priming strategy has a decreased affinity for rRNA. At least two hypotheses could explain this observation: first, the presence of oligo(dT) primers could influence priming of RNA by an unknown mechanism. Second, in this system, random and oligo(dT) primers are coupled to an RNA stretch that is necessary to create the anchoring site for the primer used in subsequent linear amplification. The sequence and length of this RNA stretch, and of the primer, are not available; therefore, we cannot exclude the possibility that they recognize some specific RNA regions, introducing a bias. The same arguments could explain the strong preference of this method towards amplification of low GC content genes.

The comparison between control and depleted libraries prepared using the SMART method revealed a greater bias, especially for the *L. lactis* sample (Additional file 2: Figures S8 and S9). This probably resulted from the low complexity of the control libraries rather than a bias induced by rRNA depletion. In fact, as the duplication rate was very high in the control libraries (at least 54%), the number of unique mappable reads and of CDSs detected also strongly decreased (66.6 and 70.9%). However, Pearson correlation between total and depleted samples was satisfactory (0.900), and the percentage of DEGs was not significant, showing that the expression patterns of the detected genes were not affected.

To verify if the complexity of the library was altered because of an insufficient initial quantity of input RNA, we prepared a library starting from 5 ng of total RNA. As expected, the duplication rate decreased to 14.4% and read results were comparable to the depleted *L. lactis* library (Additional file 1: Table S4 and Additional file 2: Figure S10). The same experiment from 5 ng total RNA was performed for the MIX and similar results were obtained. Thus, the minimal input for the SMART method should be higher than 1 ng to obtain the best performance.

### Other bacterial species

The same analyses described above were also performed for the other species to confirm the previous conclusions. Our results (Additional file 1: Tables S5, S6 and S7) confirmed the observations made for the *L. lactis* and MIX samples. Globally, TS and SMART performed best for all the species, whereas OV performances were highly variable. On *A. baylyi*, OV performed relatively well, based on CDS alignments and Pearson correlation coefficients, whereas in *B. subtilis*, the results were poor for all measures. Moreover, as in the case of *L. lactis*, the OV control libraries had lower rRNA read percentages than the SMART and TS libraries. This value varied significantly among species, with 87.6% of rRNA reads for the *E. coli* control library and only 54.8% for *B. subtilis* control library. Once again, it seems that the OV random priming disfavors rRNA retrotranscription, but at different levels according to the species. Finally, previous observations of ENC performances were confirmed in the experiments using the other bacterial species: ENC-depleted libraries showed a high percentage of rRNA reads. Similar to *L. lactis*, the ENC method correlated least well with TS. SMART had the highest correlation coefficient for all the species.

## Discussion

### Ribosomal RNA depletion does not introduce significant bias, even at very low inputs

We observed that ribosomal RNA removal treatment by Ribo-Zero is very efficient on every type of sample, as previously shown by Giannoukos et al. [21]. In contrast to this previous study, our protocol was adapted for inputs lower than those defined by the manufacturer to successfully remove rRNA from only 50 ng total RNA. Not only did rRNA reads account for less than 3%, irrespective of the downstream library preparation method, but also the depletion did not seem to introduce biases in mRNA relative abundance, as indicated by the high correlations between total RNA and rRNA-depleted libraries (Pearson coefficient >0.884).

However, the selective priming strategy adopted by the ENC method seems much less efficient. As selective primers are designed to counter-select rRNA sequences of the most common bacterial species, including our benchmark species, we predict even lower performances when the method is applied to complex environmental samples, which often comprise unknown or poorly characterized organisms.

### Technical reproducibility is high

Technical reproducibility, evaluated by Pearson correlations between replicates, was good for TS, OV and SMART (values >0.886). ENC performed less well for the *L. lactis* replicates, but not in the MIX, so we are cautious to make conclusions about its robustness. The negligible technical variation within replicates indicated that biases related to each method are also reproducible and are not influenced by the setting up of technical replicates.

### TruSeq performs well but is not adapted to “low-input” studies

The impact of the four library preparation methods on data quality was evaluated by comparing library complexity, specificity in mRNA detection, evenness of CDS coverage in individual and mixed populations, and gene expression patterns. For most of these values, TS performed best, showing the best transcriptional coverage and parameters on CDS alignments (more than 70%) and gene expression profiles. This is not surprising, as initial experiments were conducted from 4 μg total RNA. Lowering the input to 400 ng did not seem to skew the library composition (Additional file 2: Figure S11). Conversely, we tried to construct libraries with only 100 ng total RNA, but the library preparation failed or the yield was very weak (data not shown). Thus, the TS method seems to be limited by input RNA requirements and does not appear appropriate for most microbial metatranscriptomic studies.

### Encore Complete method has a higher cost per sample because of inefficient removal of rRNA

The ENC library expression profiles were comparable to those obtained with OV and SMART, and they were similar in terms of CDS coverage in both the *L. lactis* and MIX experiments. However, because of a high % of rRNA reads, ENC libraries have to be sequenced deeper to obtain sufficient transcriptome coverage. Hence, this method would introduce additional costs to metatranscriptomic studies that require an important sequencing effort to explore the complexity of these samples in depth.

### Ovation RNA-Seq system is the best solution at very low inputs but the quantitative information is biased

The OV library preparation was the simplest and most reliable in terms of library preparation workflow and final yield. It produced several micrograms of double-stranded cDNA starting from less than 0.5 ng RNA (which could be equivalent to about 50 ng if a depletion treatment was necessary). cDNA synthesis was successful even at concentrations of only a few pg (data not shown). A cDNA aliquot could then be used, after shearing, in a standard Illumina library preparation. All the preparation steps are easily amenable to automation.

Reads mapping from OV libraries clearly showed that the CDSs detected were the same found by the other methods and technical reproducibility was satisfactory. By contrast, in the MIX experiments, species distribution was significantly biased. Moreover, we detected evidence of a strong bias in gene expression profiles (r =0.589), particularly in favor of low GC content genes. Another surprising result was the relatively low percentage of rRNA reads in the total RNA control libraries. This led us to hypothesize that the random priming is not truly “random”, or that the concurrent presence of oligo(dT) or addition of RNA primer stretches could bias the RNA retrotranscription or cDNA amplification. This should be further explored by including more complex samples with GC and AT-rich species. Finally, this strategy does not provide information about strand orientation, which could be very useful for *de novo* transcriptome assembly and gene annotation. For all these reasons, we would be very cautious in applying this method for metatranscriptomic studies and, more generally, in quantitative studies.

### SMARTer is the best compromise for intermediate RNA inputs

The SMARTer method is a recent release from Clontech that aims to provide a random and stranded version of the first SMARTer Low Input library kit (oligo(dT) priming), which has been used successfully for eukaryotic single cell transcriptomic studies [26, 27]. Indeed, the Low Input oligo(dT) version is robust at very low inputs (even less than 1 ng total RNA) and we experienced this during a marine plankton metatranscriptomic study with satisfactory results (data not shown). The minimal RNA input indicated by the manufacturer for the new random stranded protocol is 1 ng RNA (depleted or total) and, to test it at the minimal input requirements, we prepared control libraries from 1 ng total RNA. Surprisingly, the final library yields were very low and the results obtained from sequencing of these libraries were not satisfactory: high duplication rates coupled to low alignment percentages suggested an insufficient library complexity. The poor efficiency was somewhat surprising but, in opposition to the oligo(dT) low-input method, this protocol does not include an exponential amplification of cDNA before Illumina library preparation. Indeed, single-stranded cDNA is directly amplified with oligonucleotides provided with the kit, which contain Illumina adaptor sequences to obtain a ready-to-sequence library. This could explain why the yields obtained with this method are different to those of the SMARTer low-input version.

The same control experiment performed starting from 5 ng of total RNA produced much better results, leading us to conclude that the minimal RNA input indicated by the manufacturer is not sufficient to preserve sample diversity, even in samples with only one bacterial species. This should be taken into account when choosing the initial RNA quantity to be depleted. Fifty ng seems to be the lower threshold to obtain sufficient library yield and satisfactory transcriptional coverage. In fact, with these inputs, the MIX libraries showed good library complexity, species representation, and gene detection and abundance. However, we could not estimate the exact depleted RNA quantity used for cDNA synthesis after RNA depletion. The rRNA quantity present in a given sample is variable, and depends on growth conditions and the state of the cell at the moment of extraction. It has been demonstrated that non-cultivated environmental bacterial cells contain less mRNA molecules than laboratory strains. For example, only ~200 transcripts are present simultaneously in a marine bacterial cell versus ~1800 mRNA molecules in exponentially growing *E. coli* [28]. mRNA yield after depletion could vary and easily be closer to 1 ng. Finally, the SMARTer method appears the most satisfying solution for metatranscriptomic studies. However, we propose that more than 50 ng of total RNA should be used to produce robust results.

### Gene expression patterns are dependent on the library construction method

One of the most relevant results of this study is the difference in gene expression patterns among the methods. We clearly demonstrated that comparison of ENC or OV data with TS or SMART would lead to false DEGs detection. By contrast, TS and SMART libraries appear to produce more accurate results, which were corroborated by qRT-PCR experiments. This emphasizes that comparison of RNA-Seq studies performed using different library protocols could be hazardous, and should be carefully evaluated. Knowledge of biases of each method, as provided in the present study, is essential to help interpret the results.

## Conclusions

In this work, we evaluated the impact of cDNA synthesis and library preparation methods on a simplified metatranscriptome. We used four well-characterized bacterial species, individually or pooled together, to mimic a controlled metatranscriptome. First, we showed that the ribosomal depletion treatment by Ribo-Zero efficiently reduces the rRNA reads and does not introduce significant bias, even at an input as low as 50 ng of total RNA. We therefore strongly suggest applying this treatment before library preparation to reduce sequencing costs, particularly in metatranscriptomics studies where deep coverage is needed.

Second, the best library preparation method was closely associated with the initial total RNA quantity used for cDNA synthesis. The TruSeq Stranded protocol produces accurate and reproducible transcriptomic patterns when RNA quantity is not limiting. However, it is not realistic to apply this method for metatranscriptomic samples, usually characterized by very low RNA quantities. Among the methods adapted to lower-input RNA, SMARTer Stranded performed adequately both in terms of library yield and data quality. It has the advantage of retaining the coding strand orientation information and it would be suitable for quantitative analysis as shown by the consistency of gene expression patterns. Importantly, we show that TS and SMART results are comparable and can be used together in DEGs analyses. Finally, with very low amounts, the Ovation RNA-Seq System V2 is the only one to guarantee the success of library preparation and sequencing. Nevertheless, this method introduces a significant bias in species abundance and gene expression patterns, probably at priming steps in reverse transcription or linear amplification. Therefore, it may not be helpful for quantitative analysis. However, it allows the production of gene catalogs with extremely low-input samples, which could not be explored otherwise. Thus, the present study provides guidelines that allow the choice of the best-fitted library preparation method for microbial metatranscriptomic studies.

## Methods

### Bacterial cultures and lysis

*E. coli* MG1655 and *B. subtilis* 168 were grown in 10 ml LB broth with shaking at 37°C to an O.D._600_ of approximately 0.5. *L. lactis* MG1363 was grown in 10 ml M17 broth supplemented with 0.5% glucose, without shaking, at 30°C to an O.D_.600_ of about 0.5. *A. baylyi* ADP1 was grown in 10 ml MAS broth at 30°C with shaking to an O.D._600_ of approximately 0.7. For each culture, 6–9-ml aliquots, corresponding to about 1 × 10^8^ cells, were harvested by centrifugation at 6000 × g for 5 min at 4°C. The supernatant was removed. Cell lysis was performed differently depending on Gram^+^ or Gram^–^ species. *L. lactis* and *B. subtilis* pellets (Gram^+^) were resuspended in 100 μL TE buffer (10 mM TrisHCl, 1 mM EDTA, pH 8) plus 10 μl lysozyme (150 mg/ml, Sigma, St Louis, MO) and 20 μl proteinase K (10 mg/ml, Qiagen, Valencia, CA). Samples were incubated at room temperature for 10 minutes with 10 sec vortexing every 2 minutes during incubation. *E. coli* and *A. baylyi* pellets were resuspended in 200 μl of preheated (95°C) Max Bacterial Enhancement Reagent (Ambion, Austin, TX) and the tubes were incubated for 4 minutes at 95°C.

### RNA extractions

RNA extractions were performed immediately after lysis by using TRIzol solution (Ambion), followed by chloroform phase separation and isopropanol/ethanol RNA precipitation. After a first quantification by Qubit 2.0 fluorometer using Qubit RNA HS Assay Kit (Life Technologies, Carlsbad, CA), 10 μg total RNA aliquots were treated with Turbo DNA-free kit (Ambion), according to the manufacturer’s instructions. After 30 minutes of incubation and final addition of the inactivation reagent, the supernatant was recovered and the Qubit 2.0 fluorometer was used to quantify the RNA.

Capillary electrophoresis on an Agilent Bioanalyzer (Agilent Technologies, Santa Clara, CA) was used to analyze approximately 3 ng of each RNA, using the RNA 6000 Pico LabChip kit. All RNA samples showed RIN values between 8 and 9.7. Assessment of the efficiency of DNAse treatment by PCR on 10 ng total RNA using strain-specific primers completed the validation of the samples. Phusion High Fidelity PCR Master Mix (Life Technologies) was used to run reactions which included a positive (fresh colony lysate in water) and a negative (nuclease-free water) amplification control. Equimolar amounts of total RNA from the four species were pooled to form the MIX sample. This was the starting material for all the synthetic metatranscriptome experiments.

### rRNA removal treatments

A Ribo-Zero Magnetic Kit for Bacteria (Epicentre, Madison, WI) was used to deplete ribosomal RNA before each cDNA synthesis by TS, SMART or OV methods. The Ribo-Zero depletion protocol was modified to be adapted to different RNA input amounts. Different total RNA inputs were depleted, varying from 50 ng up to 4 μg. The volume of magnetic beads and rRNA removal solution varied according to the RNA input as follows: 225 μl beads and 10 μl removal solution in a final volume of 40 μl for inputs >1 μg; 125 μl beads and 4 μl rRNA removal solution in a 20 μL final reaction volume for 100 ng to 1 μg inputs; 90 μl beads and 2 μl rRNA removal solution in a 20 μl reaction volume for <100 ng inputs. Except for these modifications, depletion was performed according to the manufacturer’s instructions.

Depleted RNA was purified with the RNA Clean and Concentrator–5 kit (ZymoResearch, Irvine, CA), following the procedure described for retention of >17 nt RNA fragments. RNA was eluted in 8 μl nuclease-free water in two elution steps to maximize recovery. When the total RNA input was > or equal to 400 ng, a qualitative and quantitative assessment was performed using 1 μl of depleted RNA diluted to 1:1 with water. One μl was used for quantification by the Qubit RNA HS Assay and 1 μl was run on an Agilent Bioanalyzer, using the RNA 6000 Pico LabChip kit. The Agilent profile showed a dominating small size (<200 nt) peak presumably corresponding to 5S and tRNA species. No 16S and 23S peaks were detectable. Upon Qubit quantification, recovery from 400 ng inputs was approximately 8%.

### Sequencing libraries

#### TruSeq libraries

Libraries were constructed using depleted RNA obtained from 4 μg, 400 ng and 100 ng total RNA. In the case of 4 μg total RNA input in depletion, RNA was quantified by Qubit and 30 ng of depleted RNA was used for library preparation. In the other cases, RNA was not quantifiable and the entire volume was used for library preparation. The TruSeq Stranded mRNA Sample Preparation kit (Illumina, San Diego, CA) was used. This kit includes a first poly(A) + RNA selection step by oligo(dT) beads, which may be skipped when working with rRNA-depleted RNAs, as in our case. Therefore, we introduced depleted RNAs in the preparation at the RNA fragmentation and priming step. Five μl of depleted RNA was directly added to 13 μl of Fragment, Prime, Finish mix and incubated for 8 min at 94°C for RNA fragmentation and priming. Thereafter, the libraries were prepared according to the manufacturer’s protocol without further modifications. Total RNA control libraries were constructed from 50 ng of total RNA following the same protocol modifications (poly(A) + selection step skipped).

#### Ovation libraries

Depleted RNA obtained from Ribo-Zero treatment of 50 ng total RNA was used to synthetize and amplify cDNA using the Ovation RNA-Seq System Version 2 (NuGEN, San Carlos, CA), following the manufacturer’s protocol. Control cDNA libraries were also prepared from 0.5 ng total RNA following the same protocol. cDNA yield, measured fluorometrically by Qubit DNA HS Assay, was >2 μg. An Agilent Bioanalyzer evaluated the cDNA profile using a DNA High Sensitivity LabChip kit. cDNA (500 ng) was sheared by a Covaris E210 instrument (Covaris, Woburn, MA) under the following conditions: 10% duty cycle, 5% intensity, 200 cycles per burst, 300 sec in frequency sweeping mode.

Sheared cDNA was used for Illumina library preparation by a semi-automatized protocol. Briefly, end repair, A-tailing and Illumina compatible adaptors (BiooScientific, Austin, TX) ligation were performed using the SPRIWorks Library Preparation System and SPRI TE instrument (Beckmann Coulter Genomics, Danvers, MA), according to the manufacturer’s protocol. No size selection was applied, such that most of the fragments were recovered. Twelve PCR cycles using a Platinum Pfx Taq Polymerase Kit (Life Technologies) and Illumina adapter-specific primers amplified the DNA fragments. AMPure XP beads (0.8×; Beckmann Coulter Genomics) were used to purify the libraries.

#### Encore Complete libraries

One hundred ng total RNA was used to prepare libraries for the Encore Complete Prokaryotic RNA-Seq System (NuGEN), following the manufacturer’s protocol without modifications. Briefly, after first-strand synthesis with selective proprietary primers, and second-strand synthesis with nucleotide analogs, cDNA was sheared by Covaris E210 using the same parameters described for the fragmentation of cDNA obtained using the OV method. Sheared cDNA was ligated to Illumina compatible adaptors containing the nucleotide analog in one strand. Next, the cDNA and adaptor strands containing the analog were selectively removed (Strand Selection), leaving only one cDNA strand with both adaptor sequences attached. This product was then converted into a ready-for-sequencing library by PCR amplification followed by 1× AMPure XP beads purification.

#### SMARTer libraries

Depleted RNA obtained from Ribo-Zero treatment of 50 ng total RNA was used to synthetize and amplify cDNA using the SMARTer Stranded RNA-Seq Kit (Clontech, Mountain View, CA), following the manufacturer’s protocol. After first-strand synthesis with SMARTer oligonucleotides, including partial Illumina adaptor sequences, single stranded cDNA was purified by two rounds of clean-up with 1× AMPure XP beads. The purified product was amplified by 18 cycles PCR with SeqAmp DNA polymerase and the Illumina Index Primer set, both provided in the kit. 1× AMPure XP beads were used to purify the final libraries. Control libraries were prepared from 1 ng and 5 ng total RNA.

#### Library quality control and sequencing

Libraries were quantified by qPCR using the KAPA Library Quantification Kit for Illumina Libraries (KapaBiosystems, Wilmington, MA) and library profiles were assessed using the DNA High Sensitivity LabChip kit on an Agilent Bioanalyzer. Libraries were sequenced on either an Illumina Miseq or a HiSeq2500 instrument using 150 base-length read chemistry in a paired-end mode.

#### Data quality control pipeline

After sequencing, an in-house quality control process was applied to reads that passed the Illumina quality filters (raw reads). The sequences of the Illumina adapters and primers used during the library construction were removed from the whole reads. Low-quality nucleotides (quality value <20) were removed from both ends. The longest sequence without adapters and low-quality bases was kept. Sequences between the second unknown nucleotide (N) and the end of the read were also trimmed. Reads shorter than 30 nucleotides after trimming were discarded. These trimming steps were achieved using internal software based on the FastX package [29]. The reads and their mates that mapped onto run quality control sequences (PhiX genome) were removed. Finally, the reads and their mates that mapped onto a ribosomal sequences database were removed using SortMeRNA software v 1.0 [30]. This software is designed to filter transcriptomic reads data. It contains different rRNA databases and, after treatment, splits the data into two files: rRNA reads in one file and no rRNA reads in another. The cleaned reads from the OV and SMART samples were specifically trimmed (respectively the first 12 and three bases in respect of the manufacturer’s recommendations). No specific trimming was applied to the cleaned reads from the TS and ENC samples.

All standard metrics (number of reads, number of trimmed bases, number of rRNA reads) were checked on the raw, cleaned and trimmed reads to verify the effects of each treatment. Further analyses were performed on the remaining high-quality data (cleaned reads). Data have been deposited in the European Bioinformatics Institute Short Read Archive (SRA) under accession numbers listed in Additional file 3.

#### Read mapping onto the reference CDS and statistical analyses

Each sample was mapped against its respective CDS sequences using BWA software version 0.6.1 [31] with its default parameters, allowing two mismatches in the seed (length of 35). The reference sequences were downloaded from the MicroScope website [32, 33]. For the pool, the references were put in a unique file. An estimation of the duplicated reads rate was performed on a set of 100 000 reads using markDuplicates, which is included in the Picard tools package [34]. Duplicate reads were defined as having both mates aligned at the same position on the genome. The FPKM values were calculated for each sample using the normalized reads counts for each annotated gene ((1000 × read count) ÷ (number of gene covered bases × number of mapped fragments in million)). Unmapped reads were removed, retaining only read pairs with both reads aligned to the CDS sequences. The comparison between two different samples was visualized by a scatter plot of the LOG_10_ of the FPKM. The Pearson correlation coefficient of the gene expressions between the samples were calculated using the R package version 3.0.2 [35]. The statistical analyses on the read counts were performed with the DESeq package version 1.4.1 [36] to determine the proportion of differentially expressed genes between two samples for a p-value <0.01.

#### qRT-PCR analysis

qRT-PCR quantification was performed on 10 *L. lactis* genes. Six of them were selected on the basis of read count levels in the TS experiments, from high (*eno* and *tuf*) and intermediate (*nrdE* and *secA*) to low (*gltB* and *thiD*). The other four genes, *tpx*, *gpmA*, *llmg0195* (putative NADH dehydrogenase) and *llmg2023* (universal stress protein A), were selected among the DEGs observed in the SMART, OV and ENC experiments. Primers designed using the Primer3Plus software are listed in Additional file 1: Table S8. Reverse transcription reactions were carried out on 150 ng *L. lactis* total RNA using random hexamers and Superscript II RT (Life Technologies), according to the manufacturer’s instructions. The synthesized cDNA was used as the template for qPCR reactions performed using the KAPA SYBR Fast qPCR kit (KapaBiosystems) on an Mx3005P qPCR System (Agilent Technologies). cDNA copies were quantified for each gene relative to a standard curve made from serial dilutions of purified *L. lactis* genomic DNA. Comparison of RNA-Seq and qRT-PCR quantifications was visualized by a scatter plot of the LOG_10_ of the read counts and the LOG_10_ of the qPCR quantification [37].

### Availability of supporting data

The data sets supporting the results of this article are included within this article and its additional files.

## Electronic supplementary material

Additional file 1: Table S1: Characteristics of each bacterium genome. **Table S2**: Proportions of detected genes and of covered intergenic bases. **Table S3.** Taxonomic assignation of the MIX libraries reads. **Table S4**: Sequences statistics for SMART control libraries prepared with 5 ng total RNA. **Table S5.** Sequences statistics for *A. baylyi*, *B. subtilis*, and *E. coli* libraries. **Table S6.** Proportions of detected genes and of CDS covered bases for other bacteria species libraries. **Table S7.** Pearson correlation coefficients related to *A. baylyi*, *B.subtilis*, and *E. coli* libraries comparisons. **Table S8.** Oligonucleotides used for qRT-PCR analysis. (PDF 155 KB)

Additional file 2: Figure S1: Gene expression profile of *L. lactis* samples versus MIX samples. **Figure S2.** Gene expression profile of the two replicates from *L. lactis* samples. **Figure S3.** Gene expression profile of the two replicates from MIX samples. **Figure S4.** Gene expression profile of TS versus OV, SMART and ENC methods in *L. lactis* samples. **Figure S5.** mRNA differential expression profile in *L. lactis* samples. **Figure S6.** mRNA differential expression profile in MIX samples. **Figure S7.** Scatter plots of gene expression levels detected by RNA-Seq and qRT-PCR for 10 *L. lactis* genes. **Figure S8.** Gene expression profile of depleted RNA *L. lactis* samples versus total RNA *L. lactis* samples. **Figure S9.** Gene expression profile of depleted RNA MIX samples versus total RNA MIX samples. **Figure S10**: Gene expression profile of SMART control libraries. **Figure S11**: Comparison between *L. lactis* TS libraries prepared with two different RNA inputs. (PDF 14 MB)

Additional file 3: **SRA accession numbers.** (CSV 5 KB)

## References

[1] Gilbert JA, Field D, Huang Y, Edwards R, Li W, Gilna P, Joint I (2008). Detection of large numbers of novel sequences in the metatranscriptomes of complex marine microbial communities. PLoS One.

[2] Frias-Lopez J, Shi Y, Tyson GW, Coleman ML, Schuster SC, Chisholm SW, Delong EF (2008). Microbial community gene expression in ocean surface waters. Proc Natl Acad Sci U S A.

[3] Poretsky RS, Hewson I, Sun S, Allen AE, Zehr JP, Moran MA (2009). Comparative day/night metatranscriptomic analysis of microbial communities in the North Pacific subtropical gyre. Environ Microbiol.

[4] Hewson I, Poretsky RS, Beinart RA, White AE, Shi T, Bench SR, Moisander PH, Paerl RW, Tripp HJ, Montoya JP, Moran MA, Zehr JP (2009). In situ transcriptomic analysis of the globally important keystone N2-fixing taxon Crocosphaera watsonii. ISME J.

[5] Hewson I, Poretsky RS, Dyhrman ST, Zielinski B, White AE, Tripp HJ, Montoya JP, Zehr JP (2009). Microbial community gene expression within colonies of the diazotroph, Trichodesmium, from the Southwest Pacific Ocean. ISME J.

[6] Gifford SM, Sharma S, Rinta-Kanto JM, Moran MA (2011). Quantitative analysis of a deeply sequenced marine microbial metatranscriptome. ISME J.

[7] Gifford SM, Sharma S, Booth M, Moran MA (2013). Expression patterns reveal niche diversification in a marine microbial assemblage. ISME J.

[8] Leininger S, Urich T, Schloter M, Schwark L, Qi J, Nicol GW, Prosser JI, Schuster SC, Schleper C (2006). Archaea predominate among ammonia-oxidizing prokaryotes in soils. Nature.

[9] Tveit A, Schwacke R, Svenning MM, Urich T (2013). Organic carbon transformations in high-Arctic peat soils: key functions and microorganisms. ISME J.

[10] Urich T, Lanzen A, Qi J, Huson DH, Schleper C, Schuster SC (2008). Simultaneous assessment of soil microbial community structure and function through analysis of the meta-transcriptome. PLoS One.

[11] Gosalbes MJ, Durban A, Pignatelli M, Abellan JJ, Jimenez-Hernandez N, Perez-Cobas AE, Latorre A, Moya A (2011). Metatranscriptomic approach to analyze the functional human gut microbiota. PLoS One.

[12] Stewart FJ (2013). Preparation of microbial community cDNA for metatranscriptomic analysis in marine plankton. Methods Enzymol.

[13] Carvalhais LC, Schenk PM (2013). Sample processing and cDNA preparation for microbial metatranscriptomics in complex soil communities. Methods Enzymol.

[14] Neidhardt FC, Umbarger HE, Neidhardt FC (1996). Chemical composition of *Escherichia coli*. Escherichia coli and Salmonella: Cellular and Molecular Biology, Volume 1.

[15] McGrath KC, Thomas-Hall SR, Cheng CT, Leo L, Alexa A, Schmidt S, Schenk PM (2008). Isolation and analysis of mRNA from environmental microbial communities. J Microbiol Methods.

[16] Stewart FJ, Ottesen EA, DeLong EF (2010). Development and quantitative analyses of a universal rRNA-subtraction protocol for microbial metatranscriptomics. ISME J.

[17] Yi H, Cho YJ, Won S, Lee JE, Jin Yu H, Kim S, Schroth GP, Luo S, Chun J (2011). Duplex-specific nuclease efficiently removes rRNA for prokaryotic RNA-seq. Nucleic Acids Res.

[18] Peano C, Pietrelli A, Consolandi C, Rossi E, Petiti L, Tagliabue L, De Bellis G, Landini P (2013). An efficient rRNA removal method for RNA sequencing in GC-rich bacteria. Microb Inform Exp.

[19] He S, Wurtzel O, Singh K, Froula JL, Yilmaz S, Tringe SG, Wang Z, Chen F, Lindquist EA, Sorek R, Hugenholtz P (2010). Validation of two ribosomal RNA removal methods for microbial metatranscriptomics. Nat Methods.

[20] Mettel C, Kim Y, Shrestha PM, Liesack W (2010). Extraction of mRNA from soil. Appl Environ Microbiol.

[21] Giannoukos G, Ciulla DM, Huang K, Haas BJ, Izard J, Levin JZ, Livny J, Earl AM, Gevers D, Ward DV, Nusbaum C, Birren BW, Gnirke A (2012). Efficient and robust RNA-seq process for cultured bacteria and complex community transcriptomes. Genome Biol.

[22] Adiconis X, Borges-Rivera D, Satija R, Deluca DS, Busby MA, Berlin AM, Sivachenko A, Thompson DA, Wysoker A, Fennell T, Gnirke A, Pochet N, Regev A, Levin JZ (2013). Comparative analysis of RNA sequencing methods for degraded or low-input samples. Nat Methods.

[23] Levin JZ, Yassour M, Adiconis X, Nusbaum C, Thompson DA, Friedman N, Gnirke A, Regev A (2010). Comprehensive comparative analysis of strand-specific RNA sequencing methods. Nat Methods.

[24] Sun Z, Asmann YW, Nair A, Zhang Y, Wang L, Kalari KR, Bhagwate AV, Baker TR, Carr JM, Kocher JP, Perez EA, Thompson EA (2013). Impact of library preparation on downstream analysis and interpretation of RNA-Seq data: comparison between Illumina PolyA and NuGEN Ovation protocol. PLoS One.

[25] Malboeuf CM, Yang X, Charlebois P, Qu J, Berlin AM, Casali M, Pesko KN, Boutwell CL, DeVincenzo JP, Ebel GD, Allen TM, Zody MC, Henn MR, Levin JZ (2013). Complete viral RNA genome sequencing of ultra-low copy samples by sequence-independent amplification. Nucleic Acids Res.

[26] Ramskold D, Luo S, Wang YC, Li R, Deng Q, Faridani OR, Daniels GA, Khrebtukova I, Loring JF, Laurent LC, Schroth GP, Sandberg R (2012). Full-length mRNA-Seq from single-cell levels of RNA and individual circulating tumor cells. Nat Biotechnol.

[27] Deng Q, Ramskold D, Reinius B, Sandberg R (2014). Single-cell RNA-seq reveals dynamic, random monoallelic gene expression in mammalian cells. Science.

[28] Moran MA, Satinsky B, Gifford SM, Luo H, Rivers A, Chan LK, Meng J, Durham BP, Shen C, Varaljay VA, Smith CB, Yager PL, Hopkinson BM (2013). Sizing up metatranscriptomics. ISME J.

[29] **FASTX-Toolkit** [http://hannonlab.cshl.edu/fastx_toolkit/index.html]

[30] Kopylova E, Noe L, Touzet H (2012). SortMeRNA: fast and accurate filtering of ribosomal RNAs in metatranscriptomic data. Bioinformatics.

[31] Li H, Durbin R (2009). Fast and accurate short read alignment with Burrows-Wheeler transform. Bioinformatics.

[32] Vallenet D, Belda E, Calteau A, Cruveiller S, Engelen S, Lajus A, Le Fevre F, Longin C, Mornico D, Roche D, Rouy Z, Salvignol G, Scarpelli C, Thil Smith AA, Weiman M, Medigue C (2013). MicroScope–an integrated microbial resource for the curation and comparative analysis of genomic and metabolic data. Nucleic Acids Res.

[33] **MicroScope. Microbial Genome Annotation & Analysis Platform** [http://www.cns.fr/agc/microscope/home/index.php]

[34] **Picard** [http://broadinstitute.github.io/picard]

[35] **The R Project for Statistical Computing** [http://www.r-project.org/]

[36] Anders S, Huber W (2010). Differential expression analysis for sequence count data. Genome Biol.

[37] Asmann YW, Klee EW, Thompson EA, Perez EA, Middha S, Oberg AL, Therneau TM, Smith DI, Poland GA, Wieben ED, Kocher JP (2009). 3′ tag digital gene expression profiling of human brain and universal reference RNA using Illumina Genome Analyzer. BMC Genomics.

