# Supplementary material for: Comparison of library preparation methods reveals their impact on interpretation of metatranscriptomic data
Source: BMC Genomics. 2014 Oct 20;15(1):912. doi: 10.1186/1471-2164-15-912 (PMC4213505; doi:10.1186/1471-2164-15-912)
Supplement: Supplementary file 1 — Additional file 1: Table S1: Characteristics of each bacterium genome. Table S2: Proportions of detected genes and of covered intergenic bases. Table S3. Taxonomic assignation of the MIX libraries reads. Table S4: Sequences statistics for SMART control libraries prepared with 5 ng total RNA. Table S5. Sequences statistics for A. baylyi, B. subtilis, and E. coli libraries. Table S6. Proportions of detected genes and of CDS covered bases for other bacteria species libraries. Table S7. Pearson correlation coefficients related to A. baylyi, B.subtilis, and E. coli libraries comparisons. Table S8. Oligonucleotides used for qRT-PCR analysis. (PDF 155 KB) [file 12864_2014_6604_MOESM1_ESM.pdf]

Additional File 1: Table S1. Characteristics of each bacterium genome

	GC content (%)	Genome size (Mb)	CDS cumulated size (Mb)	CDSs
***Acinetobacter baylyi* ADP1**	40	3.59	3.15	3306
***Bacillus subtilis* 168**	43	4.21	3.71	4256
***Escherichia coli* MG 1655**	50	4.63	4.08	4306
***Lactococcus lactis* MG1363**	35	2.52	2.19	2734
**MIX**	43	14.98	13.15	14602

Additional File 1: Table S2. Proportions of detected genes and of covered intergenic bases

	% detected genes				% covered intergenic bases			
	replicate 1		replicate 2		replicate 1		replicate 2	
Library name	MIX	*L. lactis* [a]	MIX	*L. lactis* [a]	MIX	*L. lactis* [a]	MIX	*L. lactis* [a]
**TS_L.L**		86.6		89.2		15.3		17.9
**ENC_L.L**		84		81		21.2		19.1
**OV_L.L**		84.6		84.7		12.9		13.1
**SMART_L .L**		88.4		87.7		18.3		18
**TS_L.L control**		90.7		91.1		18.4		18
**OV_L.L control**		92.7		85.4		12.5		11.7
**SMART_L.L control**		70.9		66.6		19.3		19.5
**TS_MIX**	76.4	63.4	72.6	73.5	16.8	15.3	17.1	15.4
**ENC_MIX**	72.8	72.7	72.7	59	18.3	19.3	18.5	19.6
**OV_MIX**	68.9	61.2	72.3	64.2	13.2	11.8	13.1	11.7
**SMART_MIX**	76.7	76.1	74.5	73.2	19	21.1	19	21.5
**TS_MIX control**	85.8	85.4	87.7	85.4	19.3	19.3	18	18
**OV_MIX control**	68.9	63.5	45	59.4	13.3	11.2	13.6	13.2
**SMART_MIX control**	64.7	63	54.8	54.6	18.2	20.1	18.2	20.5

_L.L : library prepared from *L. lactis* depleted RNA.
_L.L control : library prepared from *L. lactis* total RNA.
_MIX : library prepared from the MIX depleted RNA.
_MIX control : library prepared from the MIX total RNA.
a: for the MIX libraries, the sequences attributed to *L. lactis* were extracted and the rates were calculated for the MIX and for *L. lactis* separately.

Additional File 1: Table S3. Taxonomic assignation of the MIX libraries reads

Library name	**Reads assigned to *A. baylyi* (%)**		**Reads assigned to *B. subtilis* (%)**		**Reads assigned to *E. coli* (%)**		**Reads assigned to *L. lactis* (%)**	
	replicate 1	replicate 2	replicate 1	replicate 2	replicate 1	replicate 2	replicate 1	replicate 2
**TS_MIX**	38.4	36.5	15.05	14.59	17.24	22.2	27.86	23.65
**ENC_MIX**	35.16	35.84	14.75	14.67	19.27	20.9	27.6	25.37
**OV_MIX**	34.7	34.6	16.9	16.9	7.93	7.97	37.78	38.04
**SMART_MIX**	35.6	35.6	21.7	21.2	17.6	19.3	21.4	20.3
**TS_MIX control**	32.8	33.1	18.8	18.3	18.9	22.6	25.8	22.5
**OV_MIX control**	37	35	19.5	16.4	13.2	10.7	27.5	35.5
**SMART_MIX control**	32.9	33.2	24.5	23.6	21.5	22.4	17.8	17.6

_MIX : library prepared from the MIX depleted RNA.

_MIX control : library prepared from the MIX total RNA.

Additional File 1: Table S4. Sequences statistics for SMART control libraries prepared with 5ng total RNA

Library name	Raw reads (millions)	% rRNA [a]	Cleaned reads [b] (millions)	% mapped reads[c]	% duplication rate [d]	% detected genes [e]
**SMART_L.L control 5ng input**	18.530	95.5	0.795	63.4	14.4	88.2
**SMART_MIX control 5ng input**	18.447	93	1.242	64.7	7.21	82 (pool)<br>83.8 (*L.lactis*)

_L.L control : library prepared from *L. lactis* total RNA.
_MIX control : library prepared from the MIX total RNA.
a: proportion of rRNA reads detected in the raw reads.
b: number of sequences remaining after the data quality control pipeline applied on raw reads.
c: proportion of cleaned reads uniquely mapped on CDS sequences.
d: duplication rate estimated on 100 000 cleaned reads.
e: for the MIX libraries, the sequences attributed to *L. lactis* were extracted and the rates were calculated for the MIX and for *L. lactis* separately.

Additional File 1: Table S5. Sequences statistics for *A. baylyi* , *B. subtilis* , *E. coli* libraries

	Library name	Raw reads (millions)	% rRNA [a]	Cleaned reads [b] (millions)	% mapped reads [c]	% duplication rate[d]
	**TS_*A. baylyi***	1.770	0.15	1.762	73.7	2.59
	**ENC_*A. baylyi***	0.698	42.17	0.402	68.3	6.52
	**OV_*A. baylyi***	0.804	0.45	0.797	67.8	1.78
	**SMART_*A. baylyi***	0.707	1.16	0.693	70.3	10.28
	**TS_*B. subtilis***	0.814	0.59	0.801	61.4	8.6
	**ENC_*B. subtilis***	0.671	17.47	0.552	64.1	7.45
	**OV_*B. subtilis***	0.773	0.8	0.762	39.1	16.71
	**SMART_*B. subtilis***	0.661	0.94	0.649	60.9	18.38
	**TS_*E. coli***	2.164	0.53	2.146	69.8	4.71
	**ENC_*E. coli***	0.585	56.58	0.253	67.6	6.39
	**OV_*E. coli***	0.635	0.7	0.626	56	3.25
	**SMART_*E. coli***	0.947	2.5	0.893	58.2	14.37
**control [e]**	**TS_*A. baylyi***	21.495	91.25	1.849	72.7	4.72
**control [e]**	**OV_*A. baylyi***	31.253	66.6	10.145	74.8	2.96
**control [e]**	**SMART_*A. baylyi***	21.755	94.07	1.251	76.4	21.2
**control [e]**	**TS_*B. subtilis***	27.213	90.56	2.524	63.5	9.45
**control [e]**	**OV_*B. subtilis***	25.434	54.8	11.267	73.6	2.7
**control [e]**	**SMART_*B. subtilis***	25.431	88.3	2.865	56.7	20.12
**control [e]**	**TS_*E. coli***	28.543	93.3	1.862	70.7	4.84
**control [e]**	**OV_*E. coli***	22.536	87.6	2.696	68.9	2
**control [e]**	**SMART_*E. coli***	26.207	93.7	1.591	61.8	25.7

a: proportion of rRNA reads detected in the raw reads.

b: number of sequences remaining after the data quality control pipeline applied on raw reads.

c: proportion of cleaned reads uniquely mapped on CDS sequences.

d: duplication rate estimated on 100 000 cleaned reads.

e: library prepared from total RNA.

Additional File 1: Table S6. Proportions of detected genes and of CDS covered bases for other bacteria species libraries

	Library name	% detected genes [a]	% CDS covered bases [a]
	**TS_*A. baylyi***	98.3	82.3
	**ENC_*A. baylyi***	92.8	76.1
	**OV_*A. baylyi***	95.7	73.2
	**SMART_*A. baylyi***	96.3	73.5
	**TS_*B. subtilis***	86	65.9
	**ENC_*B. subtilis***	84.4	64.9
	**OV_*B. subtilis***	71.4	35.2
	**SMART_*B. subtilis***	84.5	53.7
	**TS_*E. coli***	95.4	83.1
	**ENC_*E. coli***	80	54.7
	**OV_*E. coli***	85.6	56.7
	**SMART_*E. coli***	88.2	63.8
**control [b]**	**TS_*A. baylyi***	98.4	94.7
**control [b]**	**OV_*A. baylyi***	93.5	80.8
**control [b]**	**SMART_*A. baylyi***	94.5	76.4
**control [b]**	**TS_*B. subtilis***	95.3	73.7
**control [b]**	**OV_*B. subtilis***	82	54.1
**control [b]**	**SMART_*B. subtilis***	86	56.2
**control [b]**	**TS_*E. coli***	95.2	82.5
**control [b]**	**OV_*E. coli***	67.4	36.1
**control [b]**	**SMART_*E. coli***	81.9	55.1

a: proportions calculated by mapping the cleaned reads on the annotations.

b: library prepared from total RNA.

Additional File 1: Table S7. Pearson correlation coefficients related to *A. baylyi* , *B.subtilis* , *E. coli* libraries comparisons

		depleted RNA vs total RNA [a]	depleted vs depleted TS [b]	total RNA vs total RNA TS [c]
A. baylyi	TS	0.901		
	ENC		0.459	
	OV	0.959	0.687	0.683
	SMART	0.987	0.851	0.864
B. subtilis	TS	0.906		
	ENC		0.394	
	OV	0.663	0.635	0.555
	SMART	0.973	0.679	0.785
E. coli	TS	0.993		
	ENC		0.57	
	OV	0.797	0.673	0.682
	SMART	0.961	0.829	0.828

Pearson correlation coefficients between:

a: the depleted RNA library and the total RNA library for each experiment.

b: ENC, OV or SMART library prepared with depleted RNA versus TS library prepared with depleted RNA.

c: ENC, OV or SMART library prepared with total RNA versus TS library prepared with total RNA.

Additional File 1: Table S8. Oligonucleotides used for qRT-PCR analysis

Highly expressed genes [a]	
llmg_0617 |*eno* Enolase	CTTGAAACAGCTGTCGGTGA
	CGTCTTCAGTTCCGTCGAAT
llmg_2050 |*tuf* Elongation factor Tu	ATACGACTTCCCAGGTGACG
	AAAGCACCAAGTGCTGAACC
moderately expressed genes [a]	
llmg_1543 |*nrdE* ribonucleotide-diphosphate reductase subunit alpha	GATGGTGGCTAACCCAGAAA
	GTTCAAGCTCACGGGCATTA
llmg_0124 |*secA* Protein translocase subunit SecA	GCTGGTCAACAAGGAGCAGT
	TGATATCTGTCCCACGACCA
low expressed genes [a]	
llmg_1183 |*gltB* glutamate synthase, large subunit	ATACGGACACCGAGGACAAG
	TCGGTACTGGAAGGAACCAC
llmg_1217 |*thiD1* phosphomethylpyrimidine kinase	GTTGAAGGGGTGATTCAAGG
	GAATAGCCCCACCTTTAGCC
Differentially expressed genes [b]	
llmg_0318 |*tpx* thiol peroxidase	GAGGGGCAAATCGTCTATAGTG
	TTAGGCTCATCCGAAAGCTC
llmg_0355 |*gpmA* 2,3-bisphosphoglycerate-dependent phosphoglycerate mutase	CGTCGTTATGCTGGTCTTGA
	AGAGCGCGTTCCAAAGTAAC
llmg_0195 putative NADH dehydrogenase	GAGCGGTCATTGTCCTTTTC
	TCTTGCAATCTCCCGAGAAC
llmg_2023 universal stress protein A	TCGTTCTTGGAGCTACTGGTC
	CCACGGAGCCGATAAAGATA

a: genes selected on the basis of TS *L.lactis* experiments.

b: genes detected as differentially expressed between TS and OV or ENC experiments.
