# Supplementary material for: Comparison of library preparation methods reveals their impact on interpretation of metatranscriptomic data
Source: BMC Genomics. 2014 Oct 20;15(1):912. doi: 10.1186/1471-2164-15-912 (PMC4213505; doi:10.1186/1471-2164-15-912)
Supplement: Supplementary file 2 — Additional file 2: Figure S1: Gene expression profile of L. lactis samples versus MIX samples. Figure S2. Gene expression profile of the two replicates from L. lactis samples. Figure S3. Gene expression profile of the two replicates from MIX samples. Figure S4. Gene expression profile of TS versus OV, SMART and ENC methods in L. lactis samples. Figure S5. mRNA differential expression profile in L. lactis samples. Figure S6. mRNA differential expression profile in MIX samples. Figure S7. Scatter plots of gene expression levels detected by RNA-Seq and qRT-PCR for 10 L. lactis genes. Figure S8. Gene expression profile of depleted RNA L. lactis samples versus total RNA L. lactis samples. Figure S9. Gene expression profile of depleted RNA MIX samples versus total RNA MIX samples. Figure S10: Gene expression profile of SMART control libraries. Figure S11: Comparison between L. lactis TS libraries prepared with two different RNA inputs. (PDF 14 MB) [file 12864_2014_6604_MOESM2_ESM.pdf]

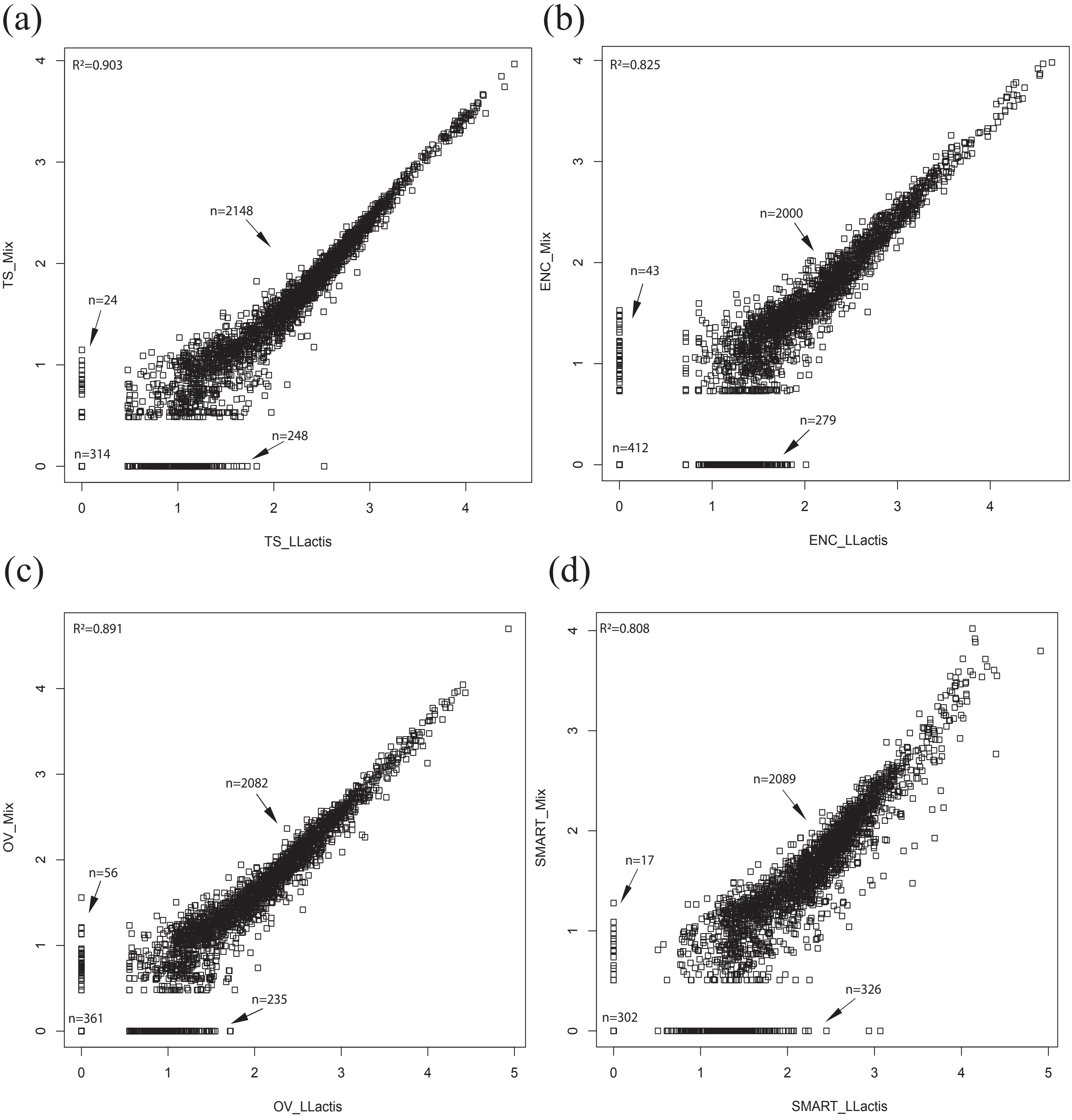

Additional File 2 : Figure S1. **Gene expression profile of *L. lactis* samples versus MIX samples.** Gene expression profile from TS_LLactis (a) and ENC_LLactis (b) OV_LLactis (c) SMART_LLactis (d) versus MIX libraries. This figure shows the log scatter plots and the coefficients of determination (R^2) obtained by comparing FPKM values for 2734 annotated CDS in *L. lactis* and 14602 annotated CDS in the mix of bacteria.

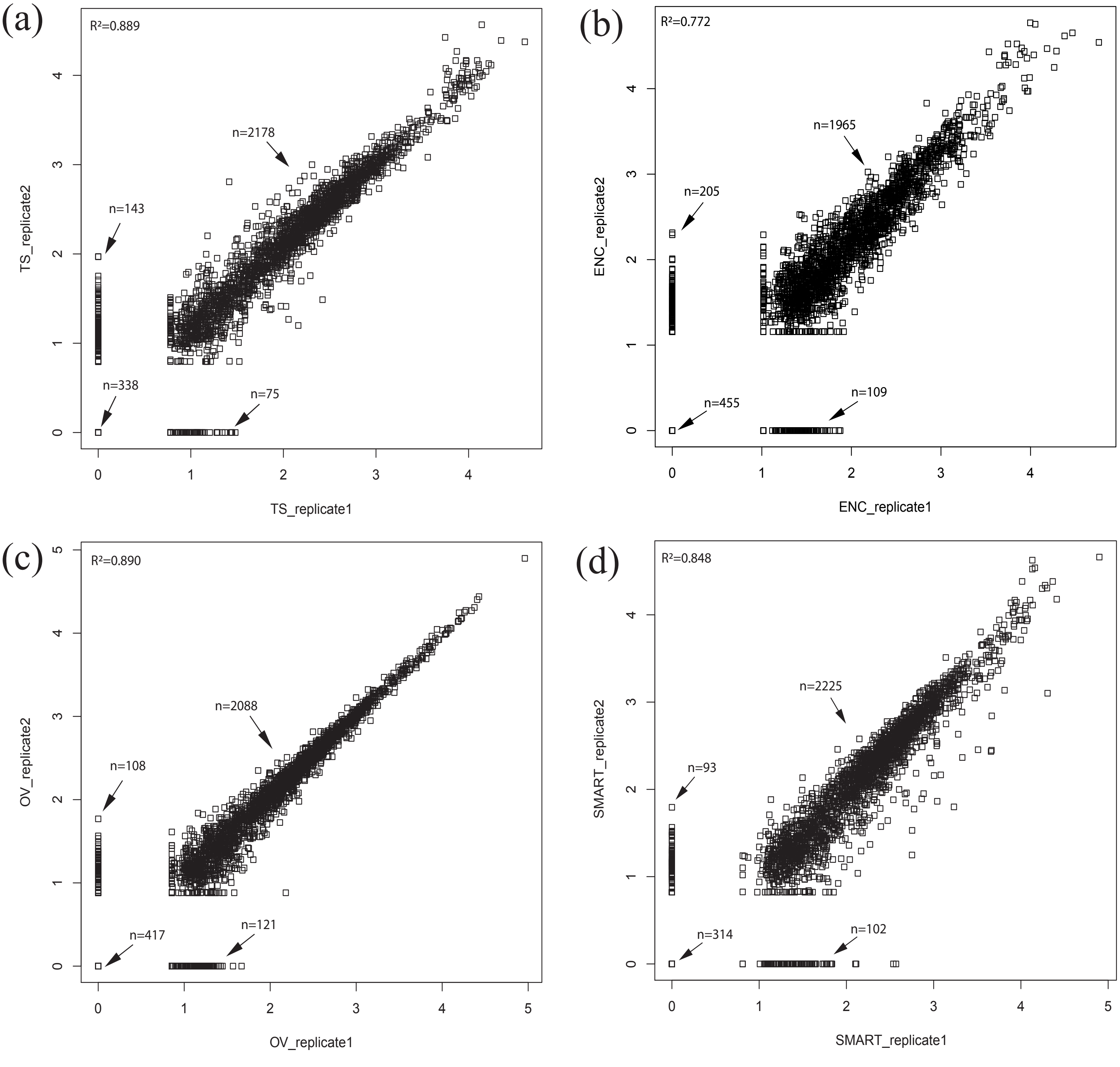

Additional File 2 : Figure S2. **Gene expression profile of the two replicates from *L. lactis* samples.** Gene expression profile from TS_LLactis and (a) ENC_LLactis (b) SMART_LLactis (c) OV_LLactis replicates samples. This figure shows the log scatter plots and the coefficients of determination (R^2) obtained by comparing FPKM values for 2734 annotated CDS in *L. lactis*.

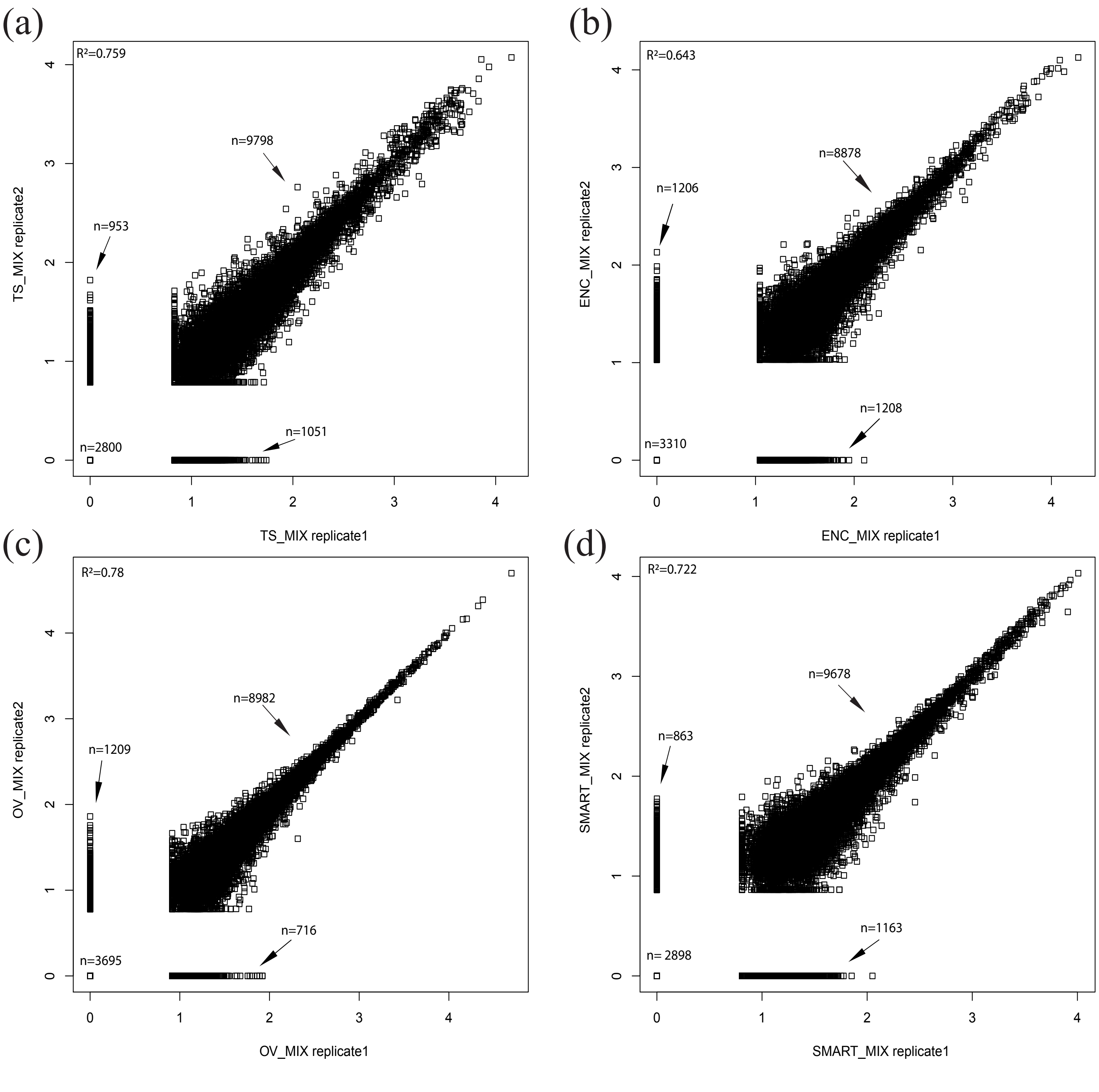

Additional File 2 : Figure S3. **Gene expression profile of the two replicates from MIX samples.** Gene expression profile from TS_mix and (a) ENC_mix (b) SMART_mix (c) OV_mix replicates samples. This figure shows the log scatter plots and the coefficients of determination (R^2) obtained by comparing FPKM values for 14602 annotated CDS in the mix of bacteria.

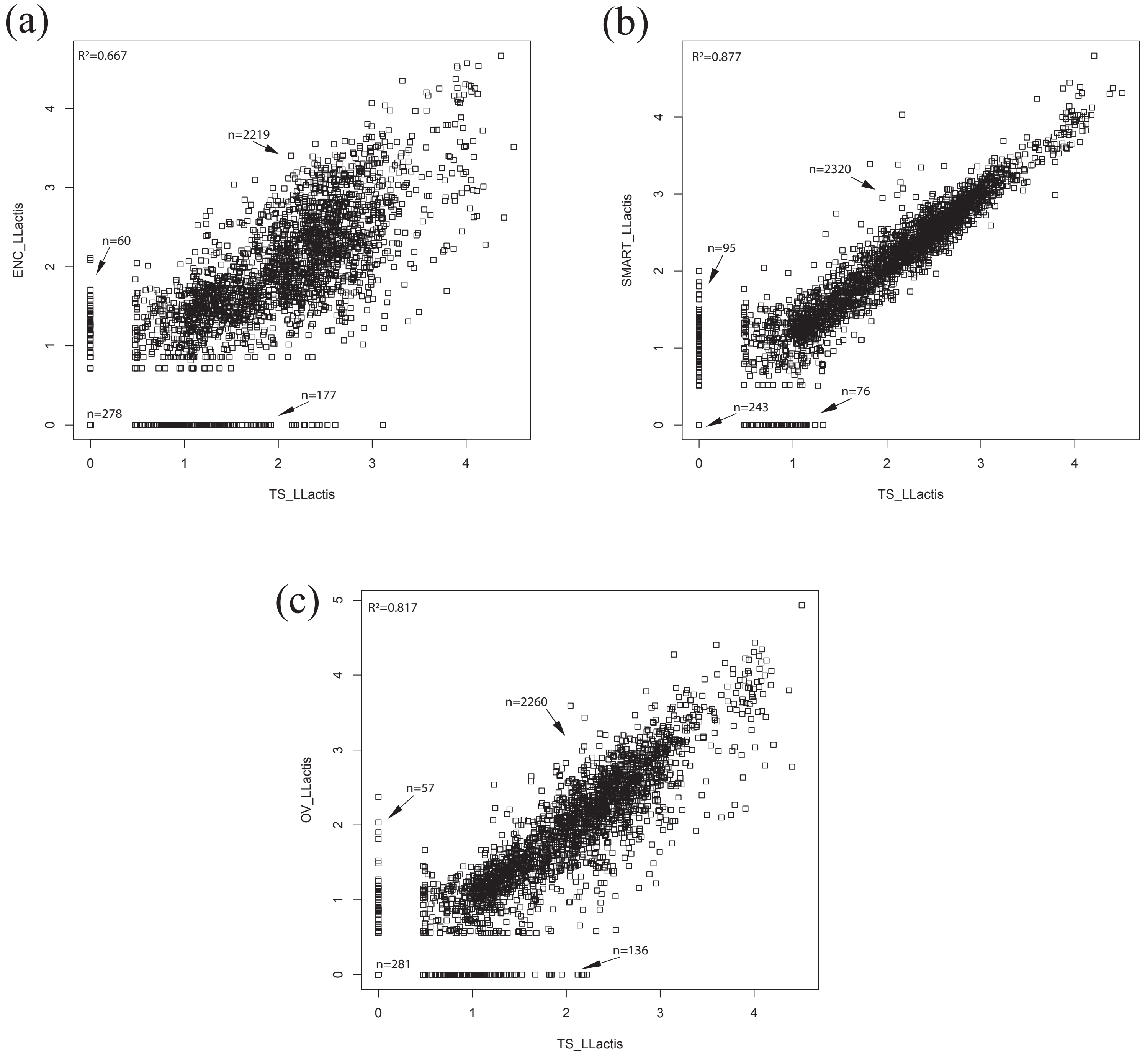

Additional File 2 : Figure S4. **Gene expression profile of TS versus OV ,SMART and ENC methods in *L. lactis* samples.** Gene expression profile from TS_LLactis versus (a) ENC_LLactis (b) SMART_LLactis (c) OV_LLactis. This figure shows the log scatter plots and the coefficients of determination (R^2) obtained by comparing FPKM values for 2734 annotated CDS in *L. lactis*.

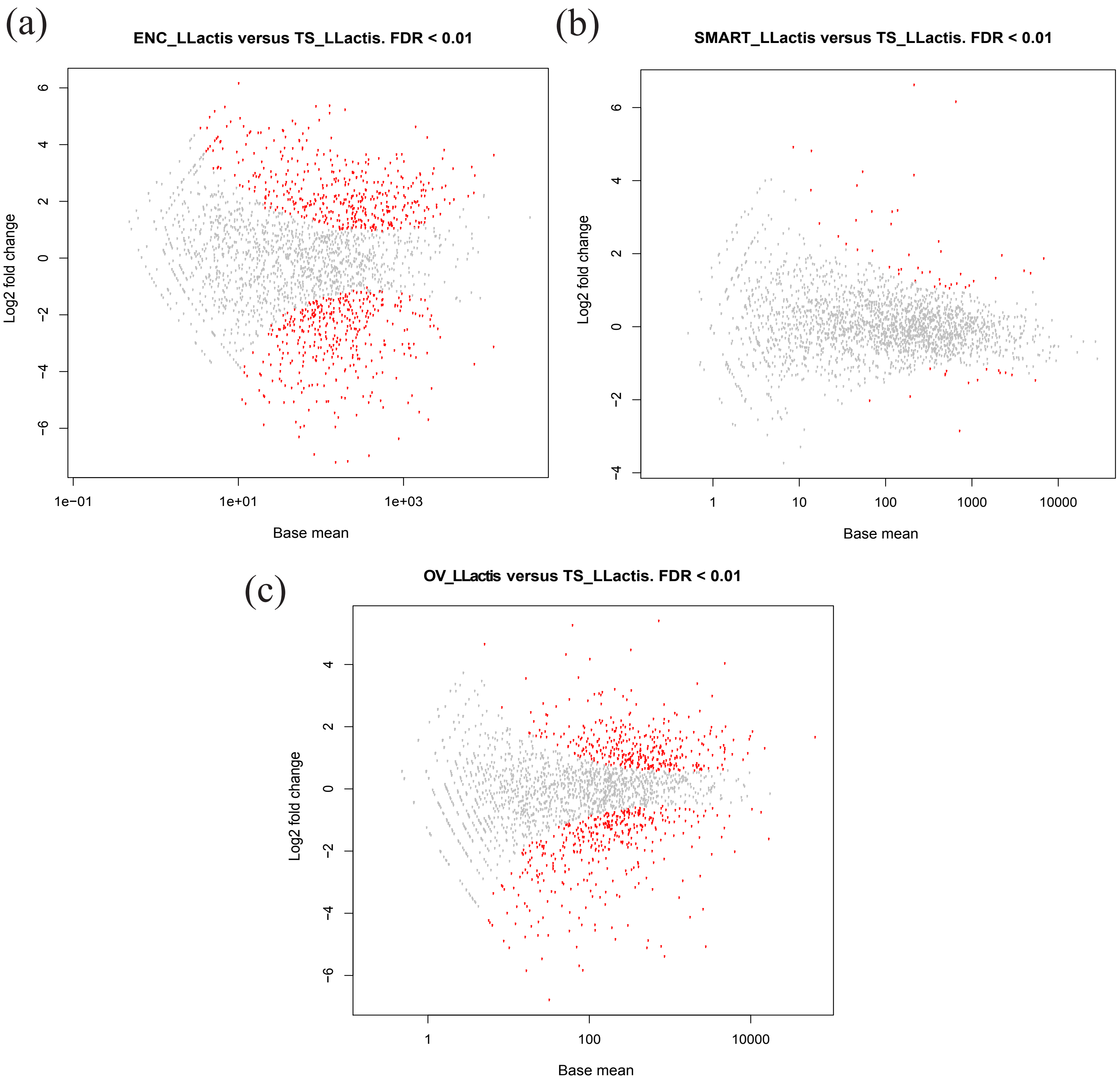

Additional File 2 : Figure S5. **mRNA differential expression profile in *L. lactis* samples.** The MAplots show the comparison of mRNA expression between (a) TS versus ENC (b) TS versus SMART (c) TS versus OV for the *L. lactis* samples. Red dots indicate mRNAs detected as differentially expressed.

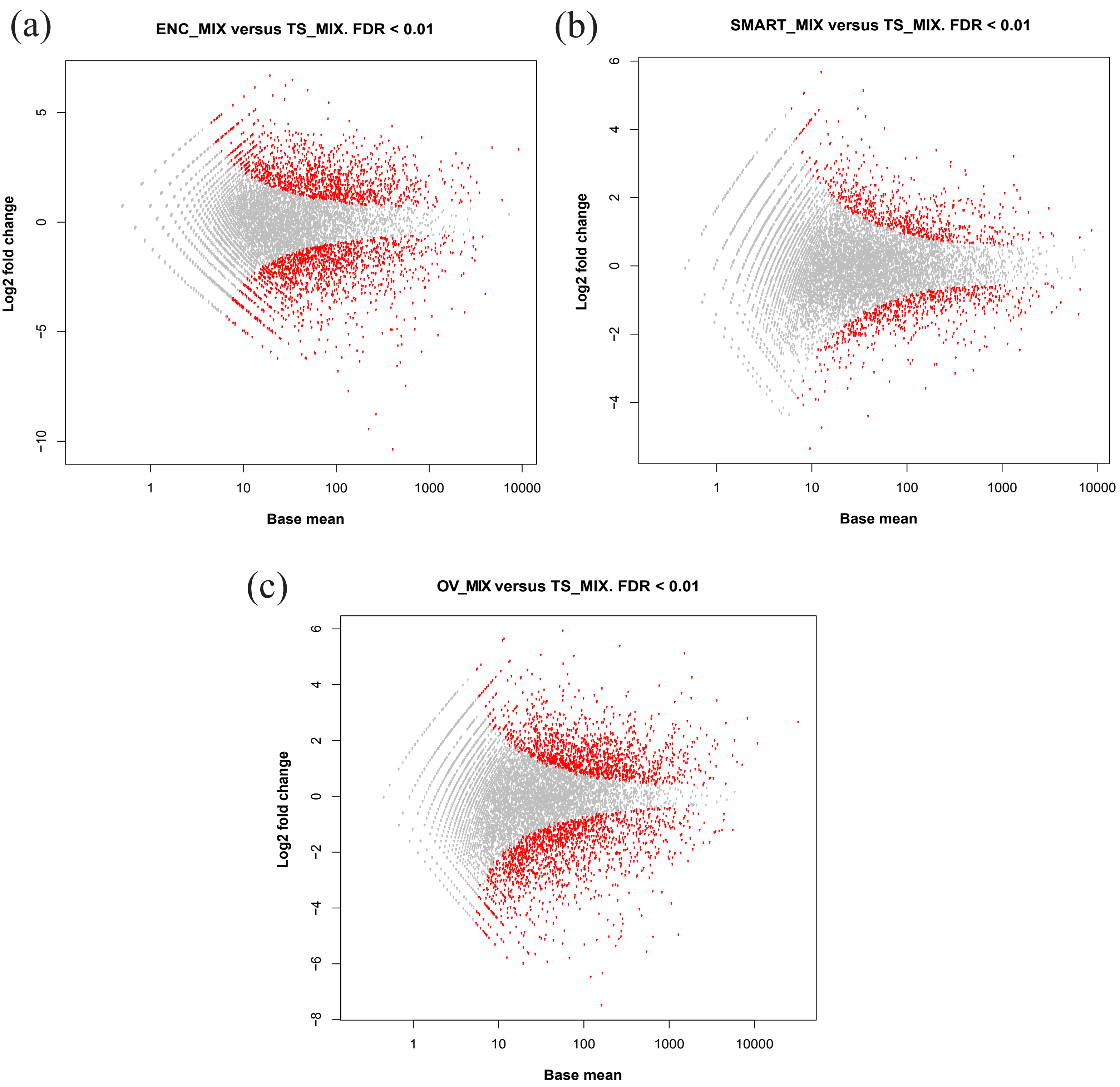

Additional File 2 : Figure S6. **mRNA differential expression profile in MIX samples.** The MAplots show the comparison of mRNA expression between (a) TS versus ENC (b) TS versus SMART (c) TS versus OV for the mix samples. Red dots indicate mRNAs detected as differentially expressed.

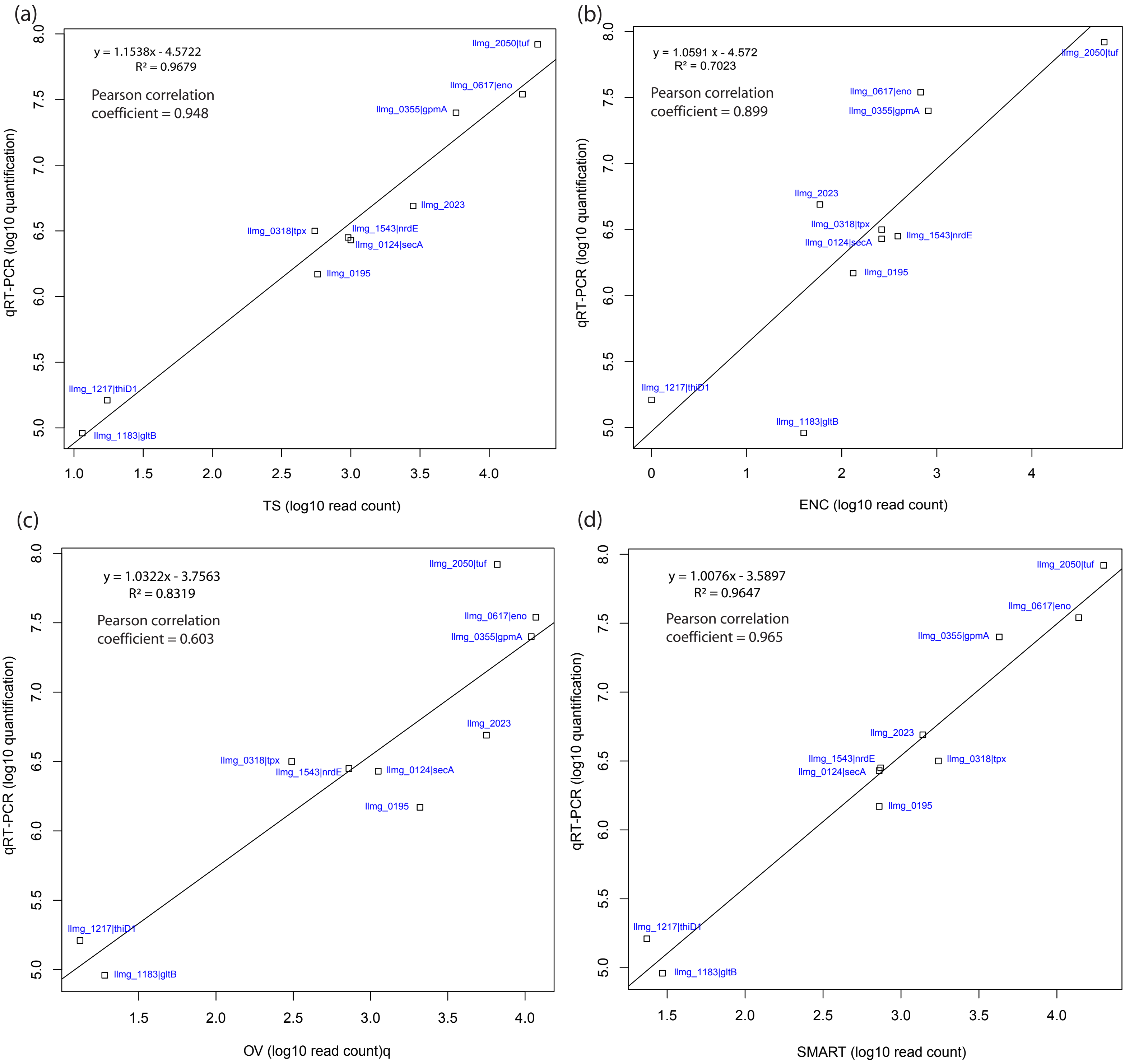

Additional File 2 : Figure S7. **Scatter plot of gene expression level measurements using RNA-Seq and qRT-PCR from 10 *L. lactis* genes.** The gene expression levels from qRT-PCR are represented by the log10 of the qRT-PCR quantification, and the gene expression levels from RNA-Seq are represented by the log10 of the read counts observed in (a) TS, (b) ENC, (c) OV, (d) SMART experiments.

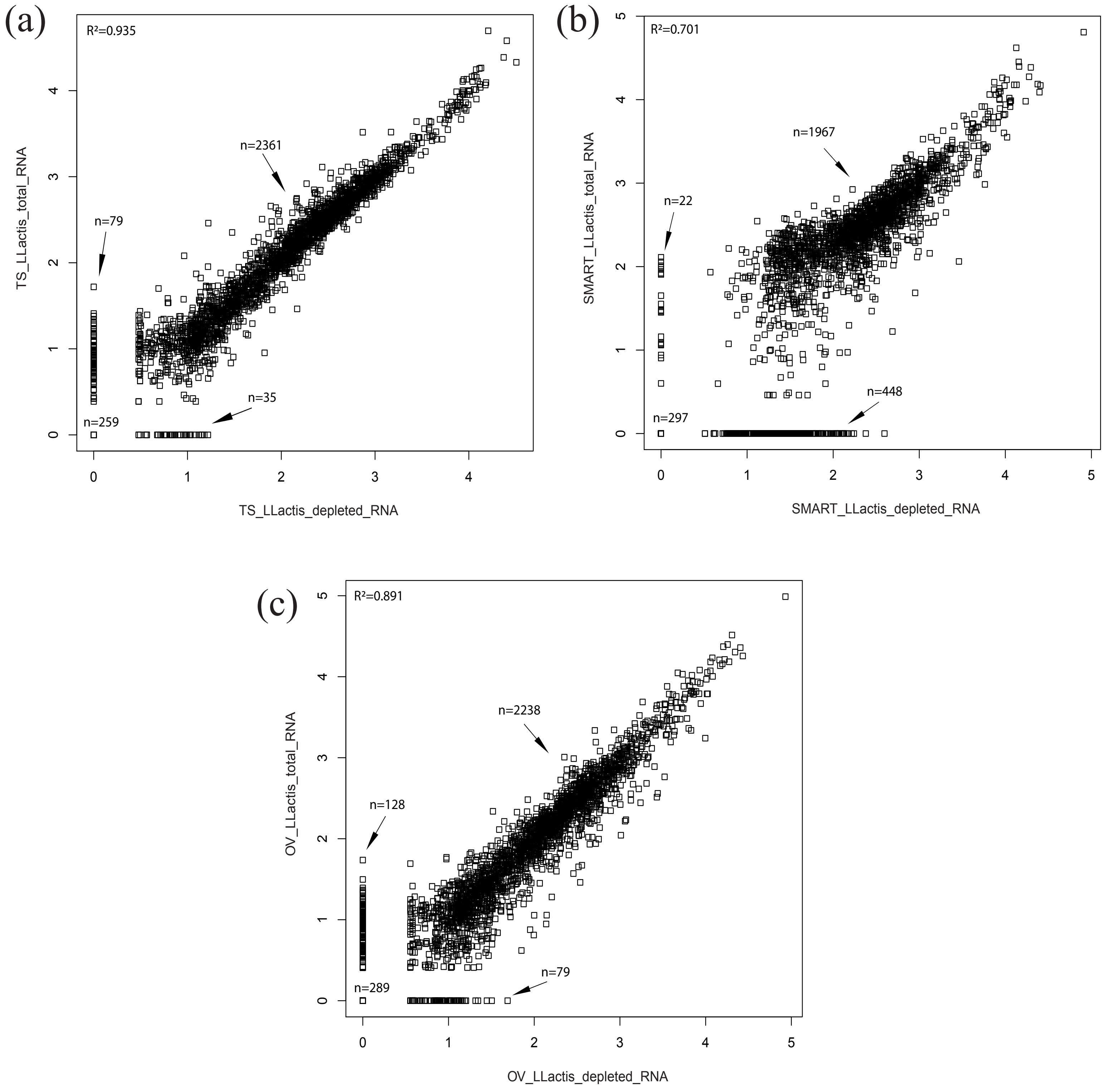

Additional File 2 : Figure S8. **Gene expression profile of depleted RNA *L. lactis* samples versus total RNA *L. lactis* samples.** Gene expression profile from TS_LLactis (a) or SMART_LLactis (b) or OV_LLactis (c) depleted rRNA sample versus total RNA sample. This figure shows the log scatter plots and the coefficients of determination (R^2) obtained by comparing FPKM values for 2734 annotated CDS in *L. lactis*.

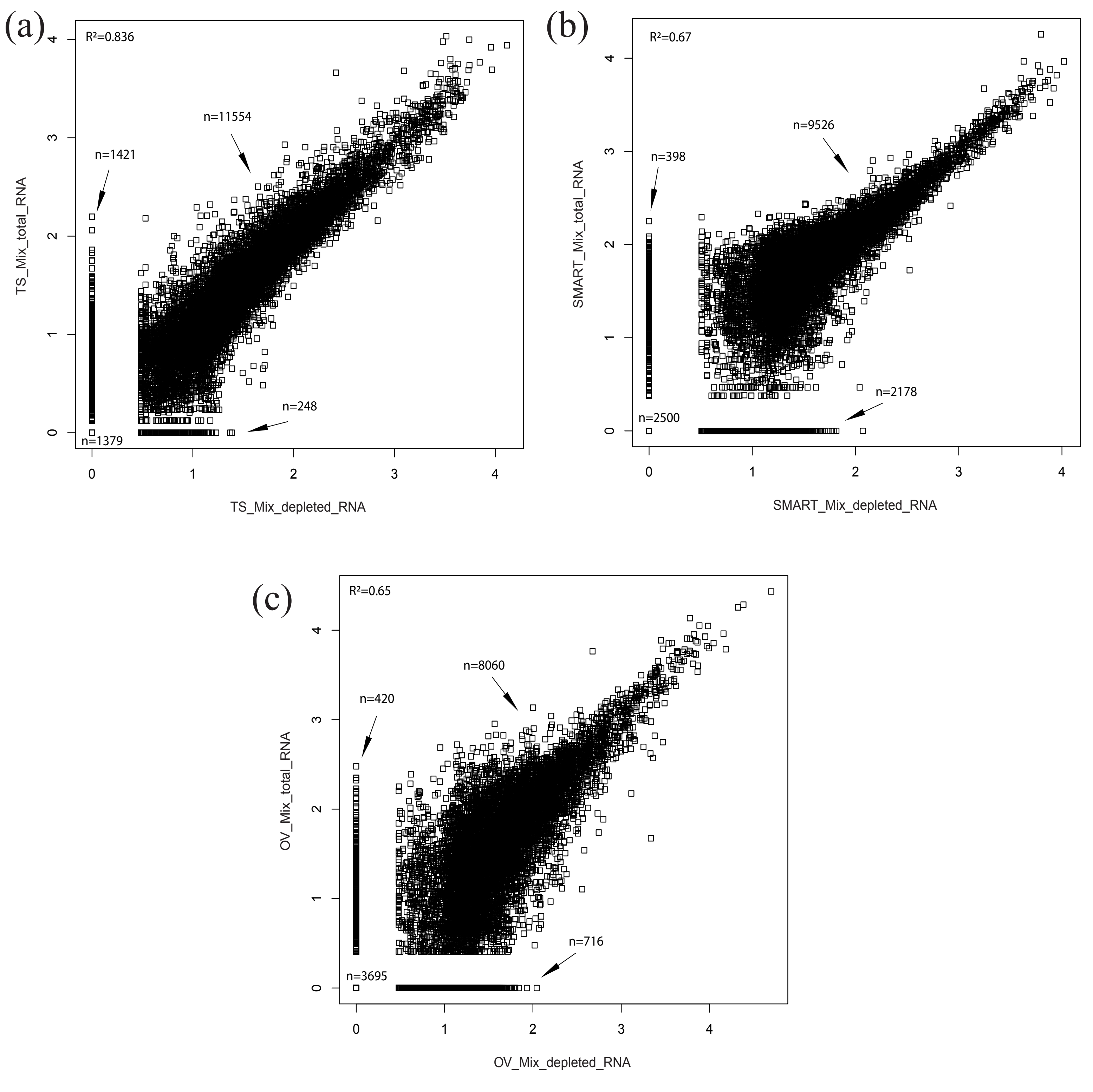

Additional File 2 : Figure S9. **Gene expression profile of depleted RNA MIX samples versus total RNA MIX samples.** Gene expression profile from TS_MIX (a) or SMART_MIX (b) or OV_MIX (c) depleted rRNA sample versus total RNA sample. This figure shows the log scatter plots and the coefficients of determination (R^2) obtained by comparing FPKM values for 14602 annotated CDS in the MIX.

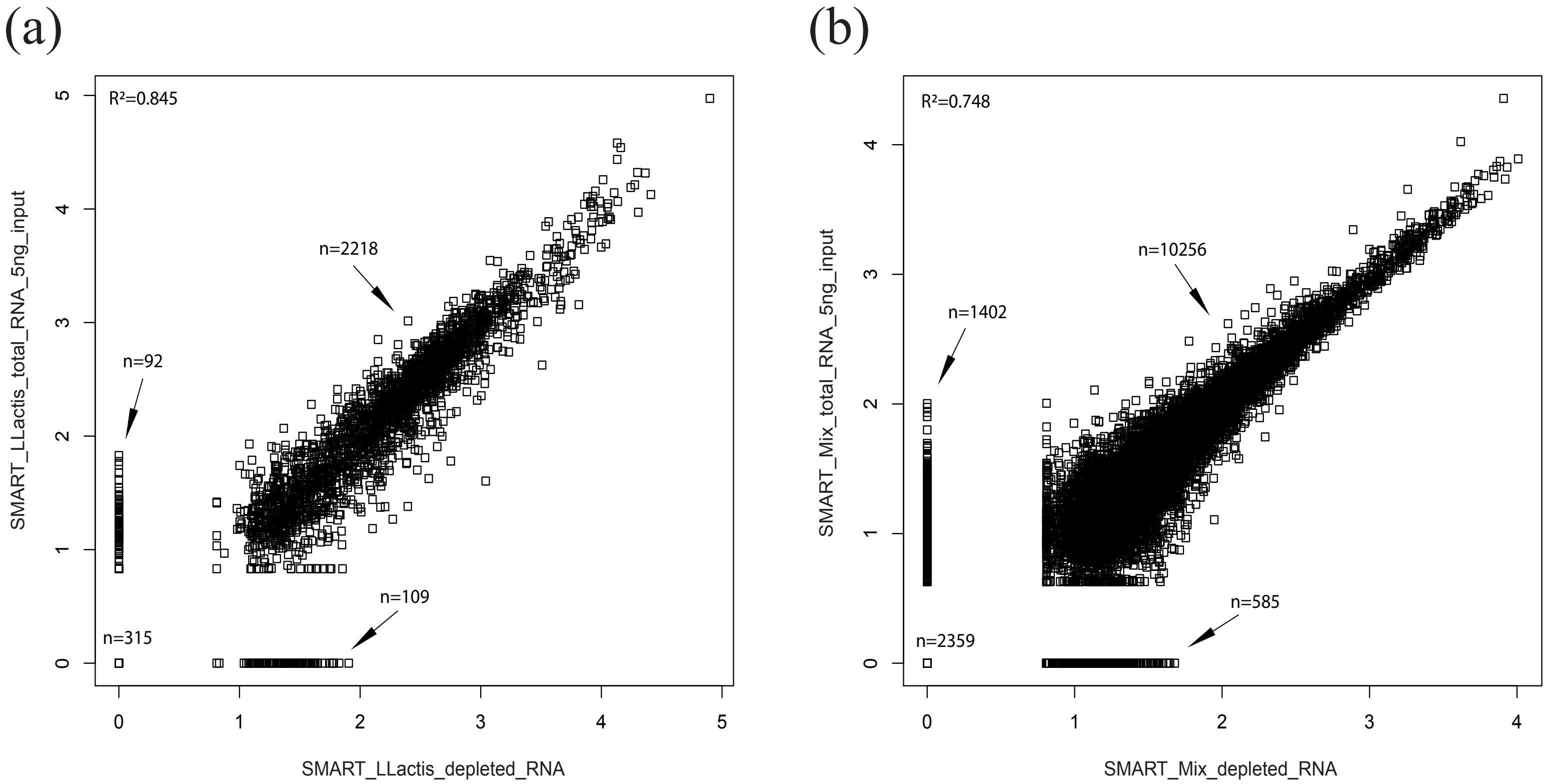

Additional File 2 : Figure S10. **Gene expression profile of SMART control libraries.** Comparison of the gene expression profile between SMART library prepared with 5ng of total RNA and SMART library prepared with depleted RNA for *L. lactis* (a) and the MIX (b).

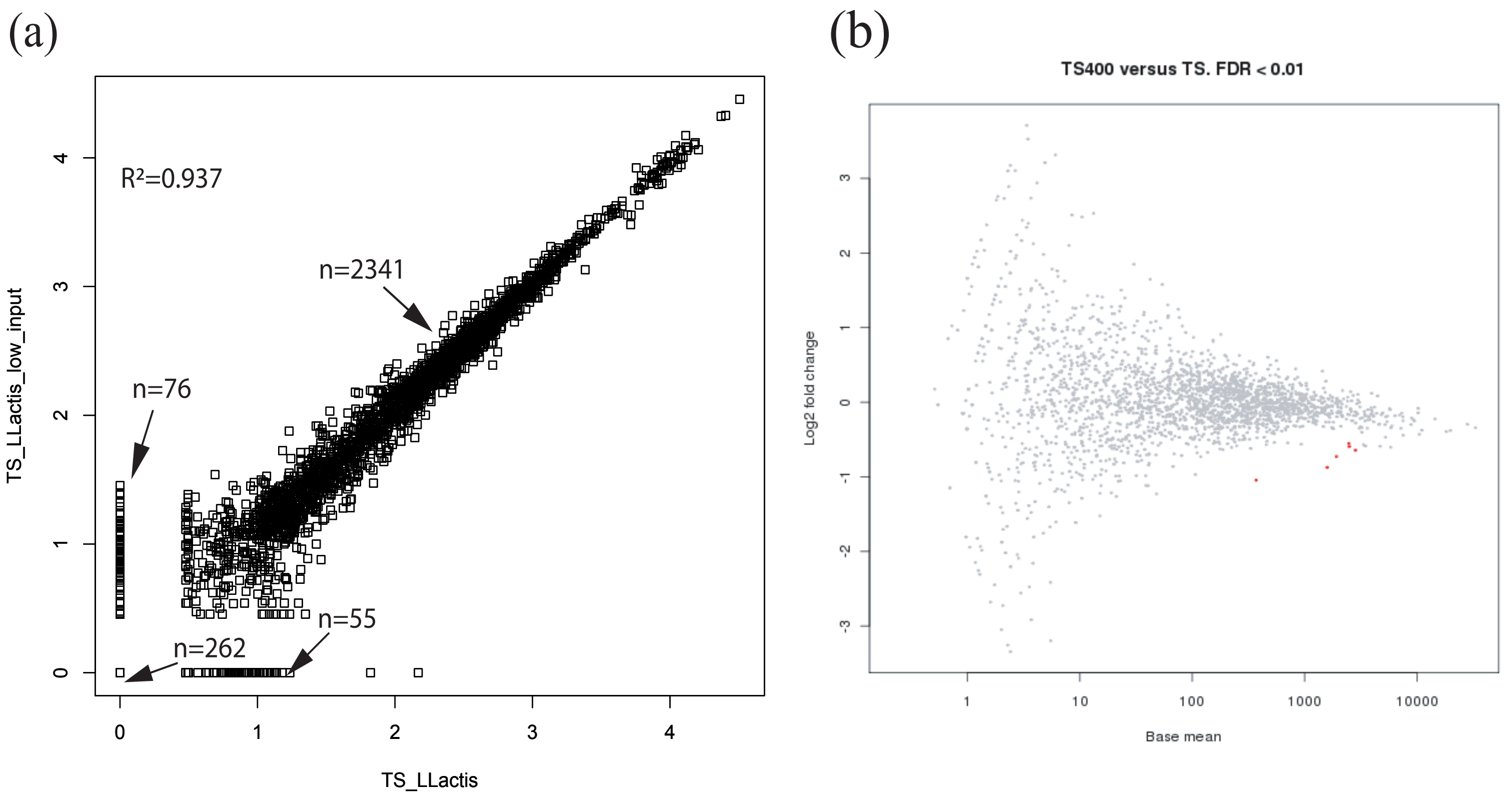

Additional File 2 : Figure S11. **Comparison between *L. lactis* TS libraries prepared with two different RNA inputs.** TS_LLactis was prepared from 30 ng depleted RNA obtained after depletion of 4µg total RNA. TS_LLactis_low_input was prepared from unquantified depleted RNA obtained after depletion of 400ng total RNA. Red dots indicate mRNA detected as differentially expressed.
